# The N-terminal extension of Arabidopsis ARGONAUTE 1 is essential for microRNA activities

**DOI:** 10.1371/journal.pgen.1010450

**Published:** 2023-03-08

**Authors:** Ye Xu, Yong Zhang, Zhenfang Li, Alyssa K. Soloria, Savannah Potter, Xuemei Chen

**Affiliations:** 1 Department of Microbiology and Plant Pathology, University of California, Riverside, California, United States of America; 2 Institute for Integrative Genome Biology, University of California, Riverside, California, United States of America; 3 Department of Botany and Plant Sciences, University of California, Riverside, California, United States of America; 4 College of Life Sciences, Shanxi Agricultural University, Taigu, China; Peking University, CHINA

## Abstract

microRNAs (miRNAs) regulate target gene expression through their ARGONAUTE (AGO) effector protein, mainly AGO1 in *Arabidopsis thaliana*. In addition to the highly conserved N, PAZ, MID and PIWI domains with known roles in RNA silencing, AGO1 contains a long, unstructured N-terminal extension (NTE) of little-known function. Here, we show that the NTE is indispensable for the functions of Arabidopsis AGO1, as a lack of the NTE leads to seedling lethality. Within the NTE, the region containing amino acids (a.a.) 91 to 189 is essential for rescuing an *ago1* null mutant. Through global analyses of small RNAs, AGO1-associated small RNAs, and miRNA target gene expression, we show that the region containing a.a. 91–189 is required for the loading of miRNAs into AGO1. Moreover, we show that reduced nuclear partitioning of AGO1 did not affect its profiles of miRNA and ta-siRNA association. Furthermore, we show that the 1-to-90a.a. and 91-to-189a.a. regions of the NTE redundantly promote the activities of AGO1 in the biogenesis of trans-acting siRNAs. Together, we report novel roles of the NTE of Arabidopsis AGO1.

## Author summary

Gene expression regulation through microRNAs (miRNAs) is widely adopted in eukaryotes. miRNAs are short RNAs ranging from 19 to 24 nucleotides, and associate with a protein named ARGONAUTE (AGO) to form miRNA-induced silencing complexes (miRISCs). A miRISC then binds to a target messenger RNA (mRNA) with sequence complementary to the miRNA, resulting in reduced expression of the target gene. AGO proteins contain four highly conserved domains: N, PAZ, MID and PIWI. Many AGO proteins also possess an N-terminal extension (NTE) region of varying lengths and sequences, of which the function is little known. In plants, most miRNAs are preferentially loaded into AGO1. In this study, we investigated the role of Arabidopsis AGO1 NTE, which is 189 amino acids (a.a.) long and highly unstructured with undefined function. By examining AGO1 mutants with truncated NTE, we show that the 91-to-189a.a. region of the NTE is essential for rescuing the developmental phenotype of an *ago1* null mutant and is required for loading miRNAs into AGO1. Moreover, we show that 1-to-90a.a. and 91-to-189a.a. regions of the NTE redundantly promote the activities of AGO1 in the biogenesis of trans-acting small interfering RNAs, a type of endogenous small RNAs that require miRNAs for their biogenesis.

## Introduction

In eukaryotes, microRNAs (miRNAs) are ~19-to-24 nucleotide (nt) long endogenous non-coding RNAs that regulate gene expression at the post-transcriptional level through sequence complementarity with target transcripts. In plants, miRNA-mediated gene silencing is essential for a broad range of biological processes, including growth, development, and responses to abiotic and biotic stresses [1,2]. *MIRNA* (*MIR*) genes are transcribed into long primary miRNA (pri-miRNA) transcripts by RNA POLYMERASE II in a manner similar to that of protein-coding genes [3]. The pri-miRNAs are successively processed by DICER-LIKE 1 (DCL1), an RNase III family enzyme, in a base-to-loop or loop-to-base manner to produce miRNA duplexes with 2-nt overhangs at the 3′ ends of each strand [4,5]. The miRNA duplexes are 2’-*O*-methylated by the methyltransferase HEN1 at the 3′ terminus of each strand [6]. The duplex is loaded into an ARGONAUTE (AGO) protein to form a miRNA-induced silencing complex (miRISC). During miRISC formation, the duplex is unwound and one strand of the duplex is selected as the miRNA (or the guide strand), while the other strand called miRNA* (or the passenger strand) is ejected [7]. Most plant miRNAs are loaded into AGO1, which prefer miRNAs with a 5’ uridine (U) [8]. A miRISC binds to a target messenger RNA (mRNA) with sequence complementary to the miRNA, leading to the degradation or translational repression of the target transcript. Studies revealed that AGO1 and miRNAs can be associated with membrane-bound polysomes, and membrane-associated AGO1 could cause both target cleavage and translational repression [9,10].

In plants, certain miRISCs, such as AGO1-miR173 and AGO7-miR390, can trigger the biogenesis of small interfering RNAs (siRNAs) from their target transcripts [11]. Upon cleavage of the target transcripts by these miRISCs, the cleaved products are converted to double-stranded RNAs (dsRNAs) by RNA-DEPENDENT RNA POLYMERASE 6 (RDR6) and the dsRNAs are processed by a Dicer protein, usually DCL4, to produce 21-nt siRNAs in a phased pattern [12,13]. Those siRNAs derived from the non-coding *TAS* loci are called trans-acting siRNAs (ta-siRNAs). In the Arabidopsis Columbia-0 (Col-0) genome, there are four families of *TAS* genes, *TAS1a/b/c*, *TAS2*, *TAS3a/b/c*, and *TAS4* [12,14–17]. The biogenesis of *TAS1a/b/c-* and *TAS2*-derived ta-siRNAs requires the AGO1-miR173 complex, and that of *TAS4*-derived ta-siRNAs requires the AGO1-miR828 complex [12,14,16]. However, for ta-siRNAs derived from the *TAS3a/b/c* loci, an AGO7-containing miRISC, AGO7-miR390, is required [18]. In addition to non-coding *TAS* loci, miRISCs such as AGO1-miR161.1 and AGO1-miR472 trigger the production of phased siRNAs (phasiRNAs) from protein-coding genes [19,20]. As in miRNA loading, ta-siRNA and phasiRNA duplexes are selectively loaded into AGO proteins based on their sequence features [8]. The ta-siRNA/phasiRNA-containing RISCs can direct target RNA cleavage and/or trigger the biogenesis of secondary siRNAs [11].

Eukaryotic AGO protein family members are highly conserved. An AGO protein contains four conserved domains: the N-terminal domain (N), which is required for small RNA duplex unwinding [21]; the PIWI/Argonaute/Zwille (PAZ) domain, which anchors the 3’ end of the miRNA guide strand [22,23]; the Middle (MID) domain, which binds the 5’ phosphate of the miRNA guide strand [24,25]; and the P-element-induced wimpy-testis (PIWI) domain, which in some AGOs, harbors an RNase H like motif that cleaves target RNA transcripts [26,27]. In Arabidopsis, AGO1, AGO2, AGO4, AGO7, and AGO10 have been shown to possess cleavage activity [7,15,28–30]. Structural studies of human AGO2 and prokaryotic AGOs show that the four domains of AGO form a two-lobed structure with a central cleft that cradles guide and target RNAs, with the N-PAZ domains composing one lobe and the MID-PIWI domains constituting the other [26,31]. The N and PAZ domains are connected by the L1 linker, while the PAZ and MID domains are connected by the L2 linker, which also connects the N-PAZ lobe and the MID-PIWI lobe [31–34].

In addition to the N, PAZ, MID and PIWI domains that are highly conserved, AGO proteins may harbor an N-terminal extension (NTE) of varying lengths and sequences, and the molecular or biological functions of this region are less understood. The NTEs of AGOs are also referred as the N-terminal coil as they were predicted to possess coil-like structures [35]. A nuclear localization signal (NLS) and a nuclear export signal (NES) are present in the NTE (1-189a.a.) of Arabidopsis AGO1 [36]. As inhibition of the EXPO1/NES-dependent protein nuclear export pathway significantly increases the ratio between nuclear and cytoplasmic AGO1, it was proposed that these signals direct the nucleo-cytosolic shuttling of AGO1 [36]. Strikingly, AGO1 with the NES sequence mutated (AGO1mNES) associated with the same miRNA cohorts as its intact counterpart. This led to the proposal that the loading of miRNAs into AGO1 takes place in the nucleus and the NES mediates the nuclear export of miRISCs [36]. However, there are other nuclear export pathways that transport AGO1 from the nucleus to the cytoplasm. The TREX-2 complex core component THP1 partners with the nucleoporin protein NUP1 at the nuclear envelope, together promoting the nuclear export of AGO1 or AGO1-miRISC [37]. Furthermore, the possibility of cytoplasmic AGO1 loading cannot be excluded. In fact, a cytoplasmically-sequestered AGO1 protein was shown to load miR165/6 *in vivo* [38].

56 mutant alleles of Arabidopsis *AGO1* have been reported in the literature, with 20 of the 56 being missense mutations, however only one of the 20 resides in the NTE [35]. *ago1-38*, a weak allele of *AGO1*, harbors a G186R mutation at the very end of the NTE region [39]. One study reported that the AGO1 protein abundance is similar between *ago1-38* and wild-type (WT), but the membrane association of AGO1 in the inflorescence tissue is reduced in *ago1-38* [40]. Furthermore, *AGO1* is under feedback regulation—*AGO1* RNA is targeted by AGO1-bound miR168 with the miR168-binding site being located in the region encoding the NTE [41]. Recent studies showed that AGO1 residues K178, K185 and K190 are required for interaction with autophagy cargo receptors ATI1 and ATI2, and mutations of these residues reduce the degradation rate of unloaded AGO1 *in vivo* [42,43]. Studies in animal AGOs show diverse functions of the NTE region. In *Caenorhabditis elegans*, WAGO-1 (a worm ARGONAUTE) and WAGO-3 are processed at their NTE by DPF-3, a dipeptidase [44]. Proteolytic activity of DPF-3 on the third and second a.a. of WAGO-1 and WAGO-3, respectively, promotes the correct sorting of 22G siRNAs into these AGOs and thus safeguard genome integrity [44]. *Drosophila melanogaster* AGO2 contains a ~400 a.a. long N-terminal glutamine-rich repeat (GRR) region, and reducing GRR copy numbers results in defects in RNAi responses and embryonic development [45].

Here, through analyzing the miRNA-related defects manifested by the Arabidopsis AGO1 NTE truncation lines, we uncover novel roles of the NTE in miRNA-mediated gene silencing. We show that loss of the entire NTE leads to a seedling-lethal phenotype resembling that of an *ago1* null allele, suggesting that the NTE is essential for the functions of AGO1. Additionally, we find that the 91-to-189a.a. region of the AGO1 NTE is essential for rescuing the morphological defects of the *ago1* null allele. Global analyses of miRNAs and their association with AGO1 revealed that the NTE, and particularly the 91–189 region, facilitates miRNAs’ loading into AGO1. Truncation of AGO1 1-to-90a.a. reduces its nuclear partitioning without affecting its profiles of miRNA and ta-siRNA association. Furthermore, we show that the biogenesis of ta-siRNAs requires the presence of either the 1-to-90a.a. or the 91-to-189a.a. region of AGO1. Taken together, our results reveal novel roles of the NTE of Arabidopsis AGO1.

## Results

### Truncation of the AGO1 N-terminal extension causes severe morphological defects

In *Arabidopsis thaliana*, the ten AGO family members can be grouped into three clades based on their phylogenetic relationship (S1A Fig) [46]. All members of the AGO1/5/10 and AGO2/3/7 clades possess NTEs longer than 130 a.a., whereas members of the AGO4/6/8/9 clade contain NTEs less than 70 a.a. long (S1A Fig). Protein sequence alignment of the NTE region of all Arabidopsis AGOs shows extremely low similarity (S1B Fig). Even AGO10, the most closely related paralog of AGO1, shares little similarity in its NTE region to that of AGO1. Furthermore, phylogenetic analysis using only the NTE regions of the ten AGOs shows that the AGO10 NTE is the most distantly related to that of AGO1 (S1C Fig). Together, the sequence analyses suggest that the NTE region of AGOs might serve AGO-specific functions.

To investigate the functions of the NTE (1-189a.a.) region of Arabidopsis AGO1, we searched for potential domains and functional motifs in this region by employing protein databases including Pfam, InterPro, and NCBI Conserved Domains Database. The 75-to-172 a.a region of Arabidopsis AGO1 was annotated as a glycine-rich_AGO1 domain by multiple protein databases. This domain appears in 117 species, all within the Magnoliopsida class (flowering plants), suggesting a unique function to flowering plants. Sequence alignment of the AGO1 NTE from six Magnoliopsida species, including *Arabidopsis thaliana* (*At*), *Arabidopsis lyrate* (*Al*), *Brassica napus* (*Bn*), *Glycine max* (*Gm*), *Oryza sativa* (*Os*), and *Zea mays* (*Zm*), shows that this glycine-rich_AGO1 domain is evolutionally conserved (S2 Fig). In addition, six RGG/RG repeats are found within the NTE region. The RGG/RG motif, which may undergo arginine methylation, is known for mediating RNA binding, protein localization, and protein-protein interactions [47]. Three of the six RGG/RG motifs are clustered at a.a. 83 to 103 in the glycine-rich_AGO1 domain, with two of them being conserved in AGO1s from the six species mentioned above except for *Oryza sativa*. One RGG/RG motif, ^59^RGG^61^, is conserved in all six species. Furthermore, glutamine (Q) content is particularly high in the NTE, with 30 Qs scattered along the region and accounting for 15.9% of the total amino acids in the NTE. This feature is conserved in the NTEs of all six inspected plant AGO1s. Next, we predicted sorting signals within the NTE that might facilitate the sub-cellular localization of AGO1. In addition to an NLS (^2^VRKRR^6^) and NES (^149^LAQQFEQLSV^158^) that were previously reported for Arabidopsis AGO1 [36], a putative NLS (^102^GGGPSSGPPQ^111^) was predicted by SeqNLS with a score of 0.737 out of 1.

**Fig 1:**
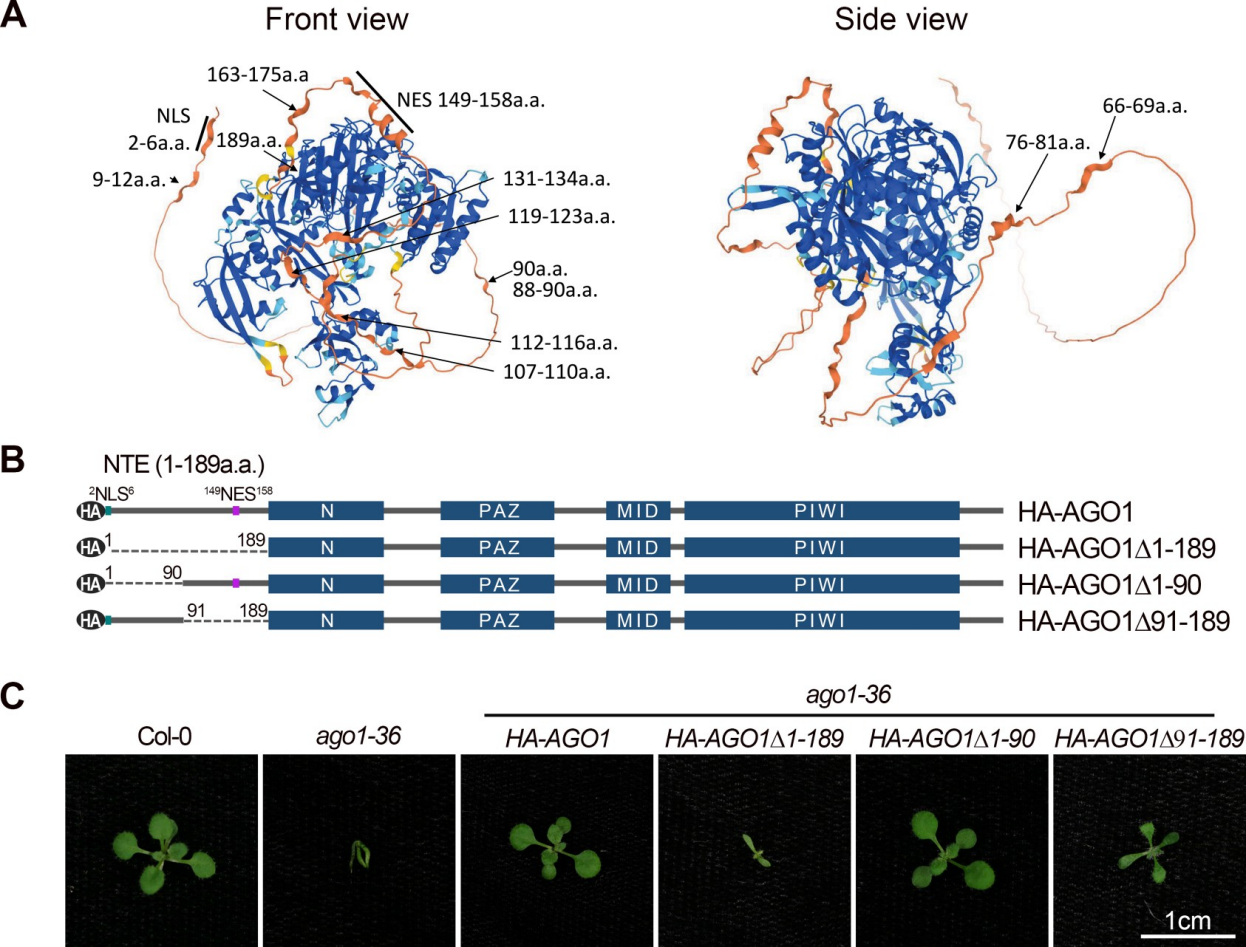
Arabidopsis AGO1 harbors an unstructured N-terminal extension. **(A)** Front and side views of a putative Arabidopsis AGO1 structure predicted by AlphaFold. The colors represent the per-residue confidence score (pLDDT), with dark blue (pLDDT > 90), light blue (90 > pLDDT > 70), yellow (70 > pLDDT > 50), and orange (pLDDT < 50) denoting high, medium, low, and very low confidence, respectively. **(B)** Schematic representation of HA-tagged Arabidopsis AGO1 and AGO1 NTE truncation forms. Blue rectangles: domains; black ovals: the HA tag; solid lines: protein sequences; dashed lines: truncated protein sequences; green: NLS; purple: NES. **(C)** Fourteen-day-old plants of Col, *ago1-36*, and *ago1-36* expressing *HA-AGO1*, *HA-AGO1Δ1–189*, *HA-AGO1Δ1–90*, and *HA-AGO1Δ91–189* transgenes. Scale bar, 1cm.

To understand how these features of the NTE affect the functions of AGO1, we tried to predict the structure of the NTE region by searching for similar protein sequences with available structural information in the Protein Data Bank using the SWISS-MODEL server. However, no protein template with significant similarity to AGO1 NTE was found. Next, we utilized AlphaFold, a machine learning tool that predicts protein structures, to get insights into the 3D structure of the NTE region (Fig 1A). The N-terminal segment (a.a 1–90) of the NTE is mostly unstructured and loosely attached to the rest of the AGO1 protein. The C-terminal segment (a.a. 91–189) of the NTE is more structured and could potentially interact with the PIWI domain of AGO1. The very C-terminus of the AGO1 NTE together with the L2 Linker is tucked in between the N and PIWI domains, and thus could potentially assist with the connection and movement between the N-PAZ lobe and the MID-PIWI lobe. The rest of the NTE C-terminus protrudes from the two lobes of AGO1, with a.a. 145–175 adopting an L shape that is close to the PIWI domain.

To further investigate how the NTE region affects the function of Arabidopsis AGO1 *in vivo*, we introduced N-terminal HA-tagged, wild-type (WT) or truncation mutants of AGO1 without the NTE region (AGO1Δ1–189), without the unstructured N-terminal segment of the NTE (AGO1Δ1–90), or without the more structured C-terminal segment of the NTE (AGO1Δ91–189) into the *ago1-36* mutant background (Fig 1B). The *ago1-36* allele contains a T-DNA insertion in the AGO1 gene at a position within the encoded PAZ domain [7]. The phenotype of *ago1-36* resembles *ago1* null alleles, such as *ago1-3* [48,49]. *HA-AGO1Δ1–90 ago1-36* line #29 and *HA-AGO1 ago1-36* line #20 were selected for downstream analysis because they had similar levels of AGO1 protein (S3 Fig). We screened over 30 transgenic lines for *HA-AGO1Δ91–189 ago1-36* and *HA-AGO1Δ1–189 ago1-36*, and all tested lines showed much higher AGO1 protein levels compared to *HA-AGO1 ago1-36 #20*. Therefore, *HA-AGO1Δ91–189 ago1-36* line #6 and *HA-AGO1Δ1–189 ago1-36* line #8 with relatively lower levels of AGO1 were selected for downstream analysis (S3 Fig). The higher levels of AGO1 proteins from *HA-AGO1Δ91–189* and *HA-AGO1Δ1–189* were likely due to the lack of regulation of *AGO1* mRNA by miR168, as the miR168 target site was removed from these transgenes. We found that both *HA-AGO1 ago1-36* and *HA-AGO1Δ1–90 ago1-36* had a WT phenotype (Fig 1C), suggesting that the N-terminal half of the NTE is largely dispensable, at least under laboratory growth conditions. *HA-AGO1Δ91–189* partially rescued the developmental phenotypes of *ago1-36* plants, whereas *HA-AGO1Δ1–189 ago1-36* seedlings resembled *ago1-36* except that the hypocotyl hook was straightened, and the cotyledons were expanded at early developmental stages (Fig 1C). This suggests that the NTE, and particularly the region of a.a. 91–189, is essential for the functions of AGO1.

### The a.a. 91–189 region of the AGO1 NTE is crucial for miRNA accumulation

**Fig 2:**
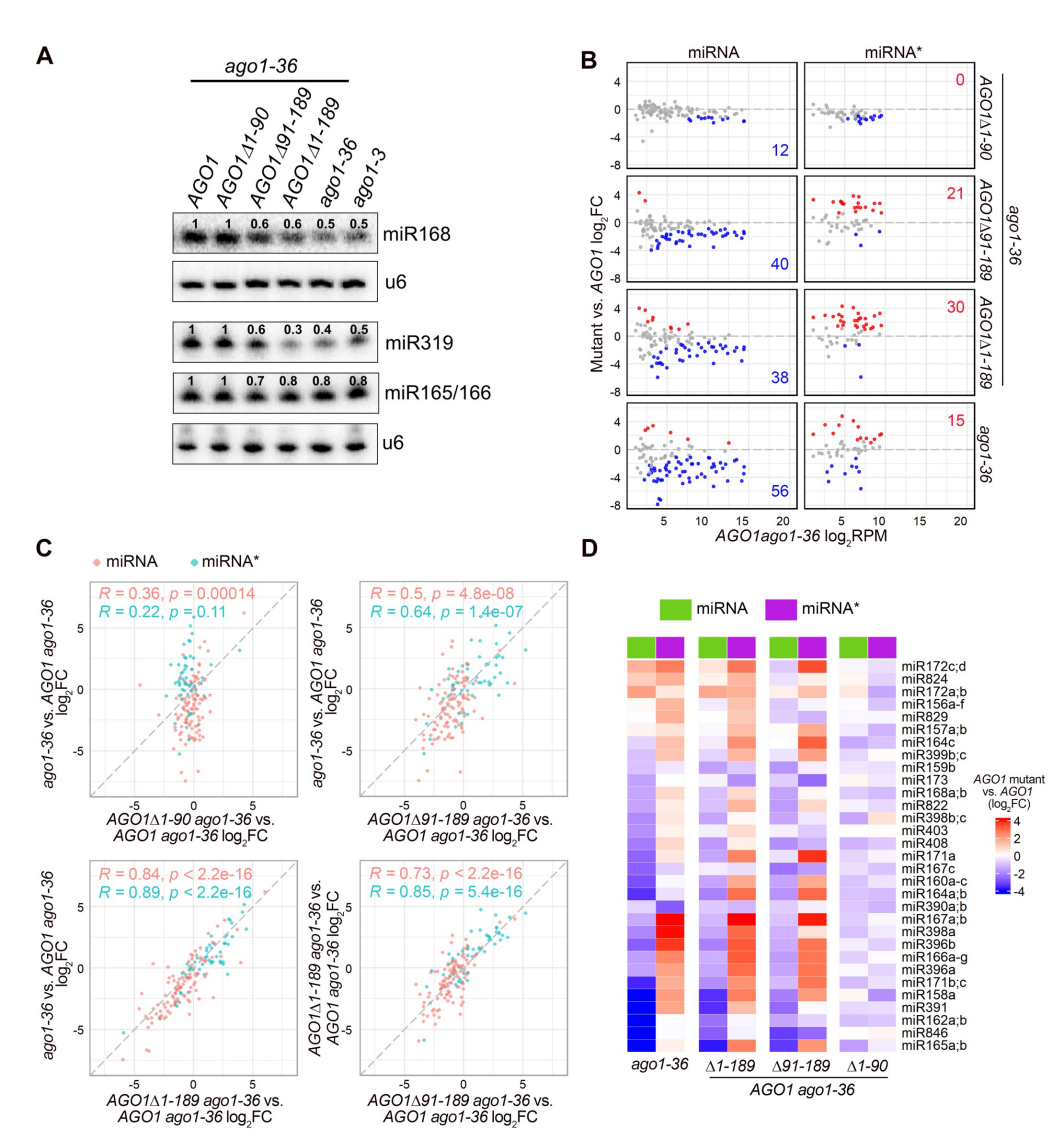
The AGO1 N-terminal extension is required for miRNA accumulation. **(A)** RNA gel blot analysis of miRNA abundance in 12-day-old seedlings of *ago1-3*, *ago1-36*, and *ago1-36* expressing *AGO1* full-length or various NTE-truncated forms. The numbers represent miRNA abundance in different genotypes relative to *AGO1 ago1-36*. The U6 blots serve as a loading control for the miRNA blots above. **(B to D)** Small RNA-seq analysis of 12-day-old seedlings. Except for *ago1-36*, all lines contain *pAGO1*:*3XHA-AGO1* or *pAGO1*:*3XHA-AGO1* NTE-truncated forms in the *ago1-36* background. Three biological replicates of each genotype were included in the analysis. **(B)** Scatter plots showing the abundance of miRNAs and miRNA*s in *AGO1 ago1-36* (X axis) and fold change between *AGO1* mutants and *AGO1* (Y axis). Red and blue dots denote miRNAs or miRNA*s with significantly increased and decreased abundance (average RPM > 2, fold change > 1.5 and adjust *P* value < 0.01), respectively. **(C)** Scatter plots comparing the log_2_(fold change) of miRNAs and miRNA*s between pairs of genotypes. Pearson’s correlation coefficients (*R*) and *p*-values are indicated for miRNAs and miRNA*s separately. **(D)** Heatmap depicting the levels of miRNAs and their corresponding miRNA*s in *AGO1* mutants relative to *pAGO1*:*3XHA-AGO1 ago1-36*. Note that only miRNAs for which the corresponding miRNA*s also passed the abundance filter (average RPM > 2) are included in the heatmap.

To determine the effects of AGO1 NTE truncation on miRNA accumulation, we performed RNA gel blot assays to determine the levels of miRNAs in seedling of *ago1-3*, *ago1-36*, and *ago1-36* expressing HA-tagged wild-type or truncated forms of AGO1. We found that compared to *AGO1 ago1-36*, the levels of three tested miRNAs were decreased in *AGO1Δ91–189 ago1-36* and *AGO1Δ1–189 ago1-36* such that they were similar to those of *ago1-36* and *ago1-3*, whereas their levels remained unchanged in *AGO1Δ1–90 ago1-36* (Fig 2A). Next, we used small RNA sequencing to examine the effects of AGO1 NTE truncations on miRNA accumulation. Principal component analysis (PCA) of the small RNA sequencing data showed that the three biological replicates of each genotype were highly reproducible (S4A Fig). The size distribution of both total small RNAs and miRNAs was similar in *ago1-36* and *ago1-36* expressing wild-type or NTE-truncated AGO1s (S4B Fig). Consistent with previous findings, total small RNAs showed a 21-nucleotide (nt) peak and a more abundant 24-nt peak, while the majority of miRNAs were 21-nt long (S4B Fig) [10,29].

Detailed analyses were carried out to compare the abundance of individual miRNAs between AGO1 NTE truncation lines and the line expressing full-length AGO1. Among a total of 428 Arabidopsis miRNAs, 104 miRNAs and 53 miRNA*s with an average level > 2 RPM (reads per million) in the small RNA-seq samples were included in the analyses. Few differentially accumulated miRNAs and miRNA*s were found between *AGO1Δ1–90 ago1-36* and *AGO1 ago1-36*, with examples being miR165a/b, miR168a/b, miR171b/c and miR390a/b (Fig 2B and S1 Table). On the other hand, more than 36% of miRNAs were decreased and over 28% of miRNA*s were increased in *AGO1Δ91–189*, *AGO1Δ1–189*, and *ago1-36* when compared to *AGO1 ago1-36* (Fig 2B and S1 Table). This result was consistent with miRNA quantification using RNA gel blot assays (Fig 2A). In accordance with the previous report on *ago1-3* [49], a null allele of AGO1, around 54% (56 out of 104) of miRNAs were significantly decreased in *ago1-36* compared to *AGO1 ago1-36*; it is likely that failure of these miRNAs to load into an AGO protein made them more vulnerable to degradation. In contrast, 7 miRNAs were upregulated in *ago1-36* compared to *AGO1 ago1-36*, for example, miR172a;b, miR866, and miR5026. These upregulated miRNAs might be preferentially sorted into and stabilized by AGO2, as 5 out of 7 upregulated miRNAs possess a 5’ adenine (A), the preferred 5’ nucleotide by Arabidopsis AGO2 [8]. In addition, 15 miRNA*s accumulated to a higher level in *ago1-36* compared to *AGO1 ago1-36*, for example, miR391* and miR393a/b*, both of which containing a 5’ adenine (A) and shown to preferentially associate with AGO2 [8]. As the majority of the upregulated miRNA*s in *ago1-36* contain a 5’ adenine (A) or guanine (G), they might be selectively loaded and protected by other AGOs when AGO1 is absent.

Comparison showed that the differentially accumulated miRNAs and miRNA*s (i.e. reduced levels of miRNAs and increased levels of miRNA*s) in *ago1-36* were highly correlated with those in *AGO1Δ1–189 ago1-36*, and with those in *AGO1Δ91–189 ago1-36*, although to a lesser extent (Fig 2C). No correlation was found for the differentially accumulated miRNAs and miRNA*s in *ago1-36* and *AGO1Δ1–90 ago1-36* (Fig 2C). These findings suggest that the AGO1 NTE (1-189a.a.), particularly the 91–189 region, is required for restoring miRNA and miRNA* levels in *ago1-36*. And as the differential accumulation of a small number of miRNAs and miRNA*s between *AGO1Δ1–189 ago1-36* and *AGO1Δ91–189 ago1-36* was not concordant, the a.a 1–90 region of the NTE might also play a minor role in miRNA accumulation.

To exam whether miRNA-miRNA* pairs are affected simultaneously in AGO1 NTE mutants, a total of 31 miRNA species with both miRNA and miRNA* passing the abundance filter (average RPM > 2) in the small RNA sequencing data were selected for evaluation. The levels of most miRNAs and their miRNA*s in *ago1-36* showed an inverse trend—while the majority of the miRNAs were downregulated, their corresponding miRNA*s were up-regulated, likely due to association with other AGOs (Fig 2D). Only a few miRNAs showed the same trend in changes in *ago1-36* to that of their corresponding miRNA*s, such as miR172a;b;c;d and miR390a;b (Fig 2D). A similar pattern was observed in *AGO1Δ1–189 ago1-36* and *AGO1Δ91–189 ago1-36*, indicating miRISC formation was likely defective for both forms of AGO1, and the a.a 91–189 region of AGO1 might be important in miRISC formation (Fig 2D).

### The a.a. 91–189 region of the AGO1 NTE is essential for miRNA and ta-siRNA loading

To test our hypothesis that the NTE region of AGO1 affects miRISC formation, we examined whether the association between miRNAs and ta-siRNAs with the NTE-truncated AGO1 is compromised. Small RNAs associated with the HA-tagged AGO1 and AGO1 mutants were immunoprecipitated (IP-ed) and sequenced. For these experiments, the *AGO1Δ91–189* and *AGO1Δ1–189* transgenes were in the *ago1-36* heterozygous background to ensure a similar cellular small RNA profile and morphological phenotypes across all tested plants. PCA analysis showed that the two biological replicates for each genotype were highly reproducible, and AGO1Δ91–189 IP and AGO1Δ1–189 IP samples were clustered together and separate from the other samples, suggesting they had small RNA profiles similar to each other yet different from AGO1 IP and AGO1Δ1–90 IP (S5A Fig). Similar to total small RNA profiles (S4A Fig), AGO1Δ1–90 IP clustered with AGO1 IP on the PC1 level, suggesting a similar small RNA binding preference between the two proteins (S5A Fig).

**Fig 3:**
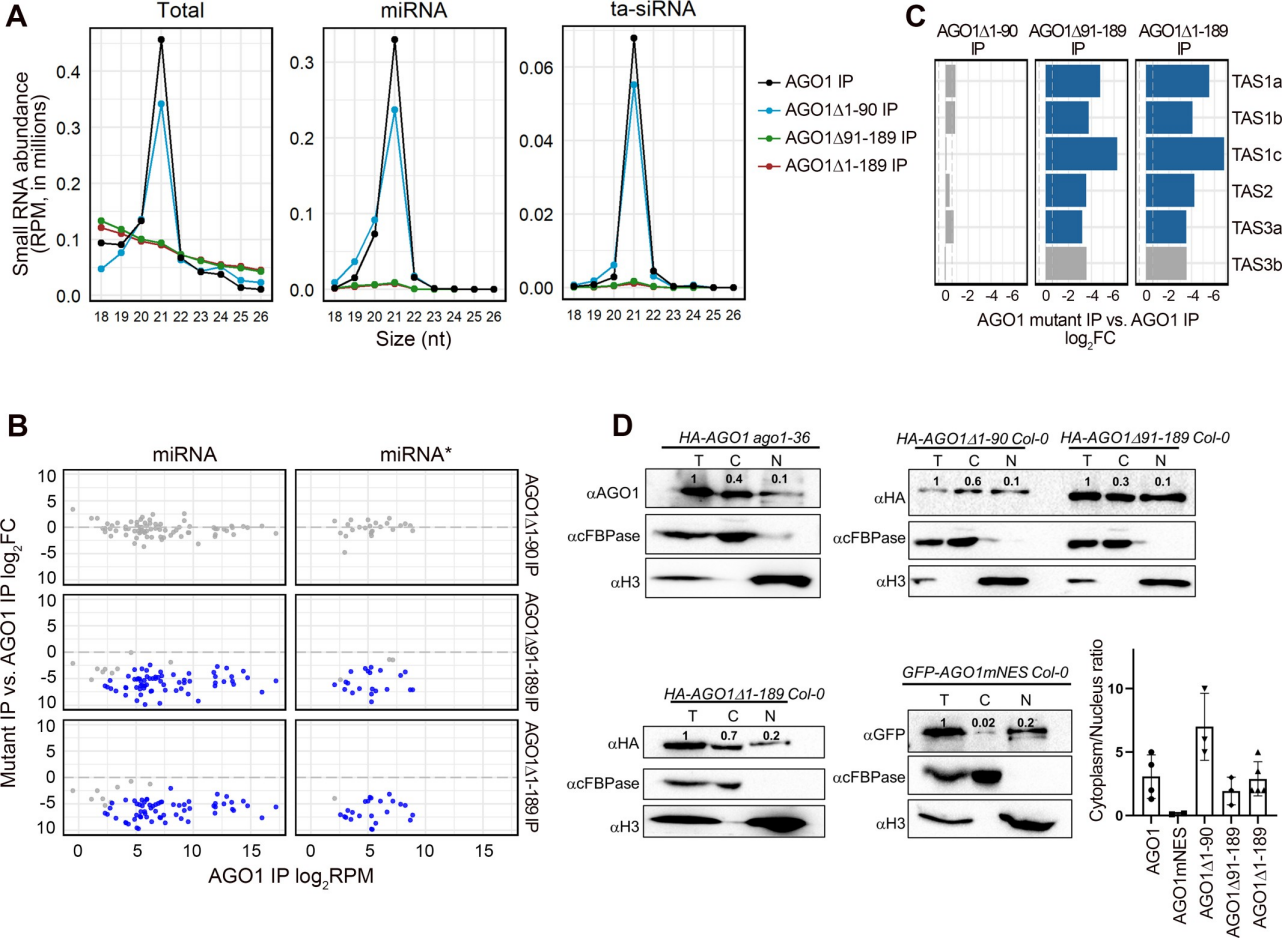
The loading of miRNAs and ta-siRNAs into AGO1 is affected by truncations of the AGO1 N-terminal extension. **(A to C)** Analysis of small RNA-seq data of AGO1-associated small RNAs. Wild-type AGO1 and AGO1Δ1–90 were in the *ago1-36* background, while other AGO1 NTE-truncated forms were in the *ago1-36/+* background. Two biological replicates of each genotype were included in the analysis. **(A)** Size (in nucleotides (nt)) distribution depicting the abundance of 18- to 26-nt small RNAs in AGO1 immunoprecipitates (IP) from plants containing AGO1 and AGO1 NTE-truncated forms. Total small RNAs, miRNAs and ta-siRNAs are shown. RPM, reads per million (see Methods). **(B)** Scatter plots showing the miRNAs and miRNA*s with significantly different levels (fold change > 1.5 and adjust *P* value < 0.01) between IP-ed AGO1 mutants and AGO1. Red dots denote miRNAs or miRNA*s with significantly higher abundance, while blue dots denote miRNAs or miRNA*s with significantly lower abundance. **(C)** Bar plots depicting the log_2_(fold change) of 21-nt ta-siRNAs between IP-ed AGO1 NTE-truncated forms and AGO1. Blue bars denote ta-siRNAs with significantly lower levels (fold change > 1.5 and adjust *P* value < 0.01). **(D)** Quantification of the nuclear-cytoplasmic partitioning of AGO1. Western blots were performed with various genotypes as indicated. Blots were probed using AGO1, HA, or GFP antibodies to detect AGO1. Histone H3 is a nuclear marker and used to quantify AGO1 in the total lysate (T) and the nuclear fraction (N). cFBPase is a cytoplasmic marker and used to quantify AGO1 in the T and the cytoplasmic fraction (C). The numbers above the bands represent relative protein levels. Bar plots summarize results of cytoplasmic/nuclear ratios of AGO1 from several experimental repeats shown here and in S7 Fig. Two replicates of wild-type AGO1 and two replicates of AGO1Δ1–90 were in the *ago1-36* background, AGO1mNES was in the Col background, while other AGO1 NTE-truncated mutants, two replicates of AGO1Δ1–90, and two replicates of wild-type AGO1 were in the Col-0 background. Error bars indicate s.d.

Consistent with previous findings [8], the size distribution of wild-type AGO1-associated total small RNAs, miRNAs, and ta-siRNAs showed a 21-nt peak (Fig 3A). Like AGO1, AGO1Δ1–90 predominantly associated with 21-nt small RNAs, however, a minor reduced preference toward 21-nt miRNAs and an increased association with 20-nt miRNAs were observed (Fig 3A). As the sequenced size of small RNAs was used in the size distribution analysis, the 20-nt miRNAs could represent a pool of annotated 20-nt miRNAs, truncated miRNAs from a larger size, and misprocessed miRNAs. We specifically analyzed the association of miRNAs with various annotated sizes with AGO1. Annotated 20-nt miRNAs constituted a slightly higher proportion in AGO1Δ1-90-associated miRNAs as compared to wild-type AGO1, suggesting that the a.a. 1–90 region of the NTE might facilitate size selection of miRNAs (S5B Fig). Strikingly, AGO1Δ91–189 and AGO1Δ1–189 completely lacked the preference toward 21-nt small RNAs, and their association with miRNAs and ta-siRNAs was minimal, suggesting that the a.a. 91–189 region of AGO1 is essential for miRNA and ta-siRNA loading (Fig 3A).

We next examined the differential association with individual miRNAs between AGO1 NTE mutants and wild-type AGO1. A total of 76 miRNAs and 24 miRNA*s were at an average level of > 2 RPM in the IP small RNA-seq samples and were included in the analysis (S2 Table). The association of miRNAs and miRNA*s with AGO1Δ1–90 was unaffected compared to wild-type AGO1, suggesting that a.a 1–90 of AGO1 have negligible effects on miRNAs loading (Fig 3B). On the contrary, more than 88% of miRNAs and 87% of miRNA*s showed reduced association with both AGO1Δ91–189 and AGO1Δ1–189 (Fig 3B), and the miRNAs and miRNA*s downregulated in the two mutants were highly correlated (S5C Fig), suggesting that the 91–189 a.a. region of AGO1 affects miRNA association globally. Furthermore, both the miRNA strand and its corresponding miRNA* strand showed reduced association with AGO1Δ91–189 and AGO1Δ1–189 (S5D Fig), implying that the reduced miRNA loading is unlikely due to defects in stand selection or miRNA* ejection. As an AGO protein stabilizes its associated miRNAs, this compromised miRNA loading could explain the severe phenotypes and the reduced abundance of miRNAs in *AGO1Δ91–189 ago1-36* and *AGO1Δ1–189 ago1-36*. The reduced association of miRNA*s with AGO1Δ91–189 and AGO1Δ1–189 also suggests that the increased accumulation of miRNA*s in *AGO1Δ91–189 ago1-36* and *AGO1Δ1–189 ago1-36* is likely due to the loading of miRNA*s into other AGOs. Similar to miRNAs, most 21-nt ta-siRNAs showed reduced levels in AGO1Δ91–189 IP and AGO1Δ1–189 IP, but not in AGO1Δ1–90 IP (Fig 3C).

Although miRNAs and ta-siRNAs were almost depleted in AGO1Δ91–189 IP and AGO1Δ1–189 IP, these proteins were still associated with small RNAs *in vivo* (Fig 3A). Wild-type AGO1 was mainly associated with 21-nt miRNAs and ta-siRNAs (Figs 3A and S6A), although it was also loaded with Pol IV-dependent siRNAs and small RNAs derived from coding genes, TEs, repeats, snRNA, snoRNA, rRNA, and tRNA (S6A Fig). We found that the proportion of rRNA- and tRNA-derived small RNAs was dramatically increased in AGO1Δ91–189 IP and AGO1Δ1–189 IP compared to wild-type AGO1 IP, while all other small RNA species showed a corresponding decrease (S6A Fig). Over 80% of the 20-, 21-, 22- and 24-nt reads from the small RNA libraries from AGO1Δ91–189 and AGO1Δ1–189 IP are derived from rRNA (S6A Fig). AGO1Δ91–189 and AGO1Δ1–189 displayed a similar loading profile toward rRNA-derived small RNAs (rsRNAs), and associated with a higher amount of rsRNAs as compared to wild-type AGO1 (S6B Fig). Consistent with the increased association with rsRNAs, examination of the 5’ end nucleotides of small RNAs associated with AGO1Δ91–189 and AGO1Δ1–189 showed a diminished preference toward uracil (U) (S6C Fig). These results suggest that a.a. 91–189 help AGO1 distinguish Dicer-dependent small RNAs from rsRNAs. Alternatively, a.a. 91–189 are critical to the loading of Dicer-dependent small RNAs, and the inability to load such small RNAs by AGO1Δ91–189 and AGO1Δ1–189 rendered them associate non-specifically with rsRNAs.

### The 91-to-189 a.a region of AGO1 facilitates miRNA loading independently of its role in nuclear-cytoplasmic shuttling

AGO1 was previously reported to shuttle between the cytoplasm and the nucleus in an NLS (a.a. 2–6)- and NES (a.a. 149–158)- dependent manner [36]. The AGO1Δ1–90 protein lacks the NLS (a.a. 2–6), and the NES (a.a. 149–158) was removed from AGO1Δ91–189, while the AGO1Δ1–189 lacks both the NLS and the NES. To investigate whether the reduced loading of miRNAs and ta-siRNAs into AGO1 NTE mutants was due to defects in nuclear-cytoplasmic shuttling, we further examined the nucleocytoplasmic distribution of AGO1 in these mutants. In addition to the NTE truncation mutants generated in this study, we also included an NES mutated AGO1 (AGO1mNES) allele that was reported before, which showed enhanced nuclear localization, intact miRNA association, and reduced ta-siRNA binding [36]. Consistent with previous findings [36,37], the cytoplasm/nucleus ratio of wild-type AGO1 was around 3:1, and this ratio was greatly reduced for the AGO1mNES protein (Figs 3D and S7). In comparison to wild-type AGO1, the levels of cytoplasmic AGO1Δ1–90 increased, supporting the nucleus-importing role of NLS (a.a. 2–6) that resides within the 1-to-90 a.a. region of the NTE. However, the reduced levels of nuclear AGO1Δ1–90 did not affect its profiles of miRNA and ta-siRNA association (Fig 3B). Interestingly, the cytoplasm/nucleus ratio of the AGO1Δ1–189 protein lacking both NLS (a.a. 2–6) and NES (a.a. 149–158) was similar to that of wild-type AGO1, suggesting that there exist mechanisms to affect the nuclear-cytoplasm shuttling of AGO1 in an NTE-independent manner. Surprisingly, AGO1Δ91–189, despite lacking the NES (a.a. 149–158) that directs AGO1’s nuclear export, was not as affected as AGO1mNES in cytoplasmic localization (Fig 3D). One possible explanation is that an unidentified motif in a.a. 91–189 of AGO1 facilitates the nuclear import. In this study, we identified a putative NLS (^102^GGGPSSGPPQ^111^) that resides in this region of AGO1, but its role in nuclear import has yet to be tested. It is worth noting that, unlike AGO1mNES, which predominantly resides in the nucleus and shows a wild-type-like miRNA-binding profile, AGO1Δ91–189 even though its nuclear accumulation is increased compared to wild-type AGO1, its miRNA association is almost depleted, suggesting that a.a. 91–189 of AGO1 regulate miRNA loading independently of its role in the nuclear-cytoplasmic partitioning of AGO1 (Fig 3D).

### The 1–90 a.a. and 91–198 a.a. regions of the AGO1 NTE function redundantly in ta-siRNA biogenesis

**Fig 4:**
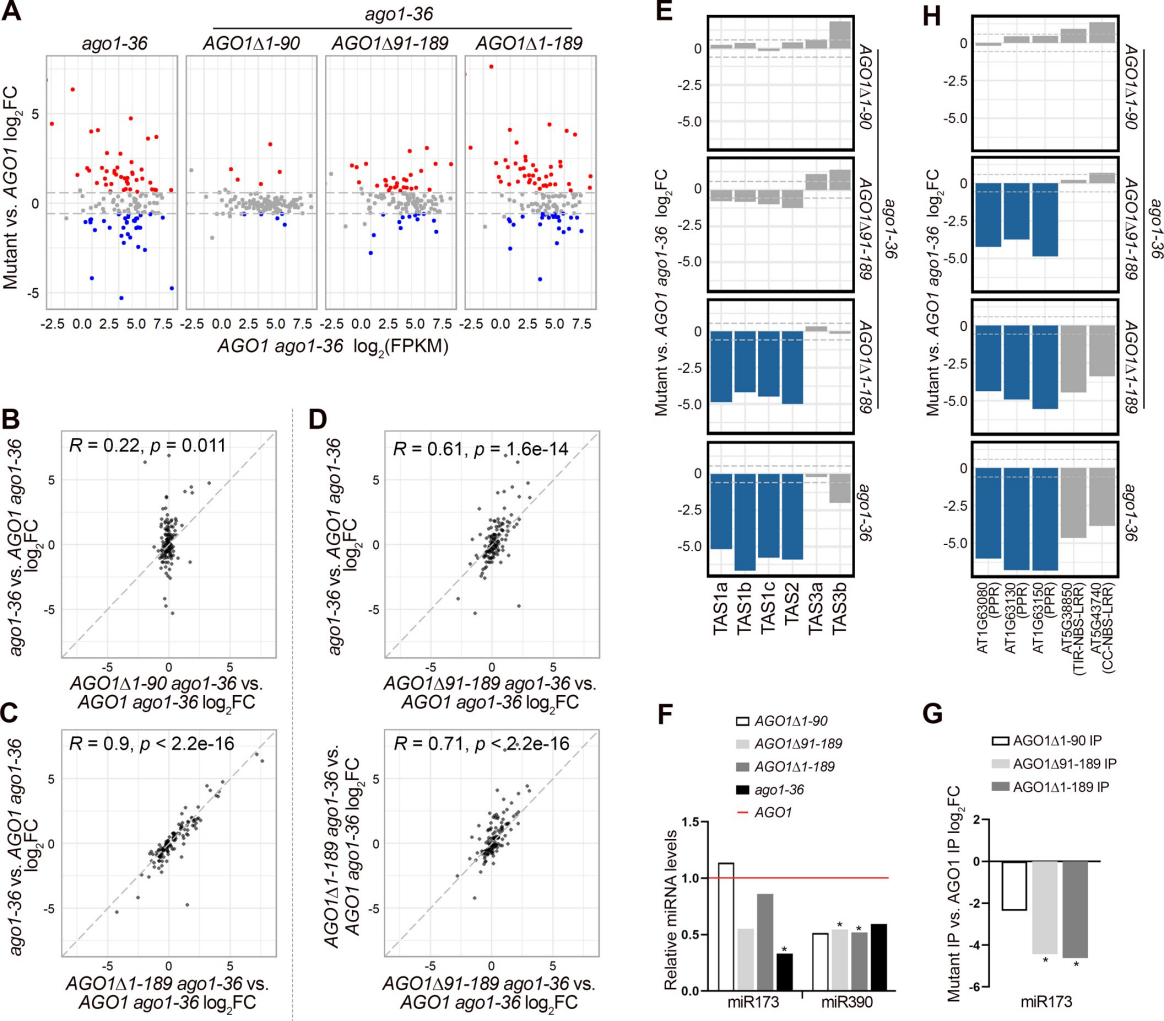
miRNA activities are affected by truncations of the AGO1 N-terminal extension as revealed by RNA-seq and small RNA-seq analysis of 12-day-old seedlings. **(A-F, H)** Wild-type AGO1 and AGO1 NTE-truncated mutants were expressed in the *ago1-36* background. Three biological replicates of each genotype were included in the analysis. **(A)** Scatter plots showing miRNA target genes with significantly different expression (fold change > 1.5 and adjust *P* value < 0.05) between *AGO1* mutants and *AGO1*. Red and blue dots denote genes with significantly increased and decreased expression, respectively. **(B-D)** Scatter plots comparing the log_2_(fold change) of miRNA target gene expression between pairs of *AGO1* mutants. Pearson’s correlation coefficients (*R*) and *p*-values are indicated. **(E)** Bar plots showing the log_2_(fold change) of 21-nt ta-siRNAs between *ago1-36* expressing various *AGO1* mutants and *AGO1 ago1-36*. Blue bars denote ta-siRNAs with significantly lower levels (fold change > 1.5 and adjust *P* value < 0.01). **(F)** Bar plots showing the relative levels of miR173 and miR390 in *ago1-36* expressing various *AGO1* mutants compared to *AGO1 ago1-36*. **(G)** Bar plots showing the log_2_(fold change) of IP-ed miR173 between AGO1 NTE mutants and AGO1. Wild-type AGO1 and AGO1Δ1–90 were in the *ago1-36* background, while other AGO1 NTE-truncated forms were in the *ago1-36/+* background. Two biological replicates of each genotype were included in the analysis. **(H)** Bar plots showing the log_2_(fold change) of 21-nt phasiRNAs between *ago1-36* expressing various *AGO1* mutants and *AGO1 ago1-36*. Blue bars denote phasiRNAs with significantly lower levels (fold change > 1.5 and adjust *P* value < 0.01).

To test whether the NTE region of AGO1 affects the activity of miRISC, we performed RNA-seq to examine the expression of miRNA target genes (S3 Table). The same RNA samples that were used in the small RNA sequencing analysis were also used for the RNA-seq analysis, including *ago1-36* and *ago1-36* expressing wild-type *AGO1* or *AGO1* NTE truncated mutants driven by the *AGO1* promoter. Three biological replicates gave highly reproducible results (S8 Fig). 130 experimentally verified miRNA target genes were investigated to identify differentially expressed genes between wild-type *AGO1* and *AGO1* NTE mutants. In comparison to *AGO1 ago1-36*, gene expression in *AGO1Δ1–90 ago1-36* was largely unaffected (Fig 4A and 4B). Around 35% of miRNA target genes were derepressed in ago*1-36* and *AGO1Δ1–189 ago1-36* (Fig 4A), and the two genotypes were highly correlated in terms of the miRNA target genes affected (Fig 4C). Interestingly, only 25% of miRNA target genes were derepressed in *AGO1Δ91–189 ago1-36* (Fig 4A), and the levels of change were lesser than their counterparts in *ago1-36* and *AGO1Δ1–189 ago1-36* (Fig 4D). The less severe molecular phenotype of *AGO1Δ91–189 ago1-36* is consistent with their better seedling phenotype compared to *AGO1Δ1–189 ago1-36* (Fig 1C), suggesting that a.a. 1–90 of AGO1 can partially rescue *AGO1Δ1–189 ago1-36*. Since miRNA association is reduced to a similar level in AGO1Δ91–189 and AGO1Δ1–189, it is possible that a.a. 1–90 of AGO1 facilitate target repression after miRNA loading.

In addition to target gene repression, certain miRNAs can trigger the biogenesis of secondary siRNAs from target loci such as *TAS1a/b/c* and *TAS2*, which produce ta-siRNAs in an AGO1-miR173 dependent manner. To determine whether the NTE of AGO1 affects ta-siRNA biogenesis, we compared the level of 21-nt small RNAs derived from the *TAS* loci and their phasing pattern. *TAS3a/b* ta-siRNAs, which require AGO7-miR390 for biogenesis, were included as a control. Strikingly, by small RNA-seq we found that ta-siRNA levels from the four AGO1-dependent loci were largely unchanged in *AGO1Δ1–90 ago1-36* or *AGO1Δ91–189 ago1-36* while the ta-siRNA levels were significantly reduced in *ago1-36* and *AGO1Δ1–189 ago1-36* (Fig 4E). In fact, the small RNA phasing patterns were similar in *AGO1 ago1-36*, *AGO1Δ1–90 ago1-36* and *AGO1Δ91–189 ago1-36* (S9A Fig). Consistently, RNA gel blot detection of *TAS1*-derived siR255 and *TAS2*-derived siR1511 showed unchanged levels in *AGO1Δ1–90 ago1-36*, and a moderate reduction in *AGO1Δ91–189 ago1-36* while their levels were significantly reduced in *ago1-36* and *AGO1Δ1–189 ago1-36* (S9B Fig). The levels of *TAS3* ta-siRNAs were unaffected in *AGO1Δ1–189 ago1-36* (Fig 4E), consistent with expectations. We further confirmed that the reduced ta-siRNA levels from *TAS1* and *TAS2* loci in *AGO1Δ1–189 ago1-36* were not due to reduced accumulation of miR173, as the levels of miR173 were largely unaffected (Fig 4F). Both AGO1Δ91–189 and AGO1Δ1–189 showed reduced association with miR173 (Fig 4G), which raised the possibility that miR173 triggered the biogenesis of ta-siRNAs in association with another AGO. However, this cannot explain the lack of ta-siRNA biogenesis in *AGO1Δ1–189 ago1-36*. A more likely scenario is that AGO1Δ91–189, despite reduced miR173 association, is sufficient to trigger ta-siRNAs production, while AGO1Δ1–189 cannot, which would suggest that a.a. 1–90 play a role in ta-siRNA biogenesis. However, ta-siRNA accumulation was not affected in *AGO1Δ1–90 ago1-36*, implying that a.a. 91–189 can also facilitate ta-siRNA biogenesis. It is likely that a.a. 1–90 and a.a. 91–189 of AGO1 redundantly enable the activities of AGO1 in ta-siRNA biogenesis.

Moreover, we noticed that phasiRNA production from protein-coding genes, such as *AGO1* and *PPR* genes, which are targeted by miR168 and miR161.1, respectively, was nearly abolished in *AGO1Δ91–189 ago1-36* but was unaffected in *AGO1Δ1–90 ago1-36* (Fig 4H), suggesting that a.a. 91–189 are required for phasiRNA production. However, small RNAs from two *NBS-LRR* genes targeted by miR472 were largely unaffected in abundance in *AGO1Δ91–189 ago1-36* (Fig 4H). Intriguingly, despite the presence of siRNAs at these two NBS-LRR genes in *AGO1Δ91–189 ago1-*36, the small RNAs were not in a phased pattern anymore (S9C Fig). The lack of phasing for *NBS-LRR-*derived siRNAs in *AGO1Δ91–189 ago1-36* but not *AGO1Δ1–90 ago1-36* suggests that a.a. 91–189 are indispensable for miRNA-triggered phasiRNA biogenesis from protein-coding genes (S9C Fig). The absence of *AGO1*-derived phasiRNAs is due to the deletion of the miR168 targeting site within the a.a. 91–189 region of the NTE (S9C Fig). We further verified that the affected phasiRNA biogenesis from *PPR* and *NBS-LRR* genes in *AGO1Δ91–189 ago1-36* was not due to reduced accumulation of their RNA transcripts (S9D Fig).

## Discussion

In this study, we show that the NTE (1-to-189 a.a.) region of AGO1 is essential for rescuing the developmental and molecular phenotypes of *ago1-36*. We further show that the a.a. 91–189 region is required for AGO1’s association with miRNAs and ta-siRNAs *in vivo* independently of its role in AGO1’s nuclear-cytoplasmic shuttling. On the other hand, the a.a 1–90 region, which contains the NLS (a.a. 2–6), is dispensable for AGO1’s association with miRNAs and ta-siRNAs *in vivo*, but plays a role in target RNA regulation. While the NTE is essential for ta-siRNA biogenesis, the presence of either a.a. 1–90 or a.a. 91–189 of the NTE is sufficient for ta-siRNA biogenesis, suggesting a redundant function of these two regions in ta-siRNA biogenesis. Below, we discuss how results from this study support these conclusions.

Proper loading into an AGO protein is essential for miRNAs to exert their roles in target gene repression. We show that miRNA association is severely and similarly compromised for AGO1Δ91–189 and AGO1Δ1–189 (Figs 3A, 3B, and S5C), suggesting a.a. 91–189 of AGO1 are required for miRNA loading. AGO1Δ1–90 IP shows a similar miRNA profile to wild-type AGO1 IP, suggesting that a.a. 1–90 of AGO1 are not required for miRNA loading (Fig 3A and 3B). A previous study shows that an NLS (a.a. 2–6) in AGO1 promotes the nuclear localization of a reporter protein (GFP-GUS) when a portion of the AGO1 NTE (a.a. 1–148) is fused to the reporter protein, while mutating an NES (a.a. 149–158) prevents the nuclear export of AGO1 [36]. It was proposed that miRNA loading into AGO1 occurs inside the nucleus, as AGO1mNES, which predominantly resides in the nucleus, shows a similar miRNA binding profile to that of wild-type AGO1 [36]. In this study we show that AGO1Δ91–189, which is unaffected or even increased in nuclear localization (Fig 3D), does not manifest a wild-type-like miRNA-binding profile (Fig 3B) as does AGO1mNES [36], suggesting that a.a. 91–189 of AGO1 are critical to miRNA loading independently of effects on the nuclear-cytoplasmic shuttling of the protein. It is possible that a.a. 91–189 of AGO1 are required for the interaction with HSP90, which is essential for miRNA loading [50]. Furthermore, we show that the reduced nuclear localization of AGO1Δ1–90 does not affect its miRNA loading or target transcripts repression (Figs 3B, 3D, and 4A), suggesting that the residual nuclear localization was sufficient to allow for miRNA loading or that miRNA loading can also occur in the cytoplasm. Indeed, miR165/6 can be loaded onto cytoplasmically-sequestered AGO1 *in vivo* [38]. Unlike AGO1mNES, which almost exclusively resides in the nucleus, approximately 30% of the AGO1Δ91–189 protein is in the cytoplasm (Fig 3D), suggesting a motif that promotes nuclear import could be in the deleted region. An NLS (^102^GGGPSSGPPQ^111^) that resides in the a.a. 91–189 region of AGO1 was predicted in this study. Mutating both NLS (a.a. 2–6) and the putative NLS (a.a. 102–111) might create a cytoplasm-exclusive AGO1 that is suitable to test our hypothesis.

The 1-to-90 a.a. region of AGO1 is not required for rescuing the developmental defects of *ago1-36* or for miRNA loading, however, we found it can facilitate target repression and ta-siRNA biogenesis. We show that the association of miRNAs with AGO1Δ91–189 and AGO1Δ1–189 is almost abolished (Fig 3A and 3B), whereas miRNA target gene repression is less affected in *AGO1Δ91–189 ago1-36* compared to *AGO1Δ1–189 ago1-36* (Fig 4A and 4D), implying that a.a. 1–90 of AGO1 assist in target transcript cleavage. For ta-siRNA biogenesis from *TAS1* and *TAS2* loci, both *AGO1Δ1–90 ago1-36* and *AGO1Δ91–189 ago1-36* resemble *AGO1 ago1-36* in terms of ta-siRNA levels and phasing patterns, while *AGO1Δ1–189 ago1-36* and *ago1-36* are defective in ta-siRNA biogenesis (Figs 4E and S9A). Although miR173, the trigger miRNA for the biogenesis of *TAS1a/b/c-* and *TAS2-*derived ta-siRNAs, was significantly reduced in AGO1Δ91–189 IP compared to wild-type AGO1 IP (Fig 4G), it is unlikely that other AGOs associate with miR173 to trigger ta-siRNA biogenesis when AGO1 loading is compromised, as miR173-dependent ta-siRNA biogenesis is greatly affected in *ago1-36* (Fig 4E). We hypothesize that only a small amount of AGO1-miR173 is required to trigger the biogenesis of ta-siRNAs in *AGO1Δ91–189 ago1-36*. Together, these observations suggest that a.a. 1–90 of the NTE are sufficient for ta-siRNA biogenesis.

Another interesting observation was that *AGO1Δ91–189 ago1-36* was defective in phasiRNA biogenesis, as evidenced by reduced siRNA abundance at three *PPR* genes and lack of siRNA phasing at two *NBS-LRR* genes (Figs 4H and S9C). The trigger miRNAs for ta-siRNA and phasiRNA biogenesis, such as miR173, miR161.1, and miR472, all show reduced association with AGO1Δ91–189 (Fig 4G and S2 Table), but why is AGO1Δ91–189 capable of ta-siRNA biogenesis but not phasiRNA biogenesis from protein-coding genes? One possibility is that these two processes require different auxiliary proteins, and a.a. 91–189 are required for specific protein-protein interactions. Alternatively, these two processes might take place at distinct subcellular locations, and a.a. 91–189 are required for the localization of AGO1 to one of the subcellular compartments.

## Methods

### Plant materials and growth conditions

All *Arabidopsis thaliana* lines in this study were in the Columbia (Col) ecotype. Seeds of *ago1-36* (SALK_087076) were obtained from the ABRC collection. Seeds were surface-sterilized with 75% ethanol and stratified in water at 4°C for 2 days then transferred to 1x Murashige–Skoog (MS) medium. All plants were kept in a growth chamber at 22°C under full-spectrum white light and long-day conditions (16 h light / 8 h dark).

*ago1-36* and *ago1-36/+* expressing *pAGO1*::*3XHA-AGO1*, *pAGO1*::*3XHA-AGO1Δ1–90*, *pAGO1*::*3XHA-AGO1Δ91–189*, and *pAGO1*::*3XHA-AGO1Δ1–189* were generated in this study. To construct the plasmids of the above transgenes, a 1648bp *AGO1* promoter (including the 5’ UTR) and the 399bp 3’ UTR were amplified from genomic DNA, the full length *AGO1* coding region and NTE-truncated *AGO1* were amplified from cDNA, and the 3xHA tag was amplified from pGWB615 [51]; these fragments were then cloned into pEarleyGate 301 [52] (predigested by XbaI and NcoI) using the NEBuilder HiFi DNA Assembly Cloning Kit (NEB). All clones were validated by sequencing. Sequences of the primers are listed in S4 Table.

### Small RNA gel blot analysis and sequencing

RNA isolation and RNA gel blot analysis of small RNAs were performed as described [10]. 5–10 μg of total RNA was isolated from 12-day-old Arabidopsis seedlings using TRI Reagent (NRC), resolved in 15% urea–PAGE gels and transferred to NX membranes (Amersham Hybond-NX). MiRNAs were detected with 5’ end ^32^P-labeled antisense DNA oligonucleotide probes. Sequences of the DNA oligonucleotide probes in this study are listed in S4 Table. Hybridization signals were analyzed by phosphorimager (Typhoon 9410, GE).

Small RNA sequencing and data analysis were performed as described [10]. 25 μg of total RNA was extracted from 12-day-old Arabidopsis seedlings and resolved in a 15% urea-PAGE gel, and small RNAs of 15- to 40-nt were isolated from the gel. Small RNA libraries of gel-purified small RNAs or small RNAs acquired from AGO1 immunoprecipitation (as described below) were constructed using the NEBNext Multiplex Small-RNA Library Prep Set for Illumina (E7300). The libraries were sequenced on an Illumina HiSeq X Ten, and the resulting data were analyzed using an in-house pipeline pRNASeqTools v.0.8 (https://github.com/grubbybio/pRNASeqTools). The three-prime-end adapter sequence (AGATCGGAAGAGC) was trimmed from raw reads, followed by filtering to retain 18- to 42-nt reads using cutadapt v3.0 [53]. Trimmed reads were then aligned to the Arabidopsis genome (TAIR10) using ShortStack v.3.8.5 [54] with parameters ‘-bowtie_m1000 -ranmax 50 -mmap u -mismatches 0’. For total small RNAs, reads were normalized against total 18- to 42-nt reads minus 45S rRNA reads (RPM, Reads Per Million Reads). For small RNAs acquired from AGO1 immunoprecipitation, reads were normalized against IP-ed 18- to 42-nt reads (RPM). Differential comparison of small RNAs was conducted by DESeq2 v1.30.0 with fold change of 1.5 and adjusted *P* value < 0.01 as the parameters [55]. Annotation of miRNA and miRNA* sequences was obtained from miRbase v21 (http://www.mirbase.org/). For miRNA length distribution and small RNA composition analysis, reads that match perfectly or with a 1-nt shift on either end from the annotated sequence were assigned to the miRNA. For miRNA differential expression analysis, only reads that match perfectly to the annotated sequence were assigned to the corresponding miRNA. For ta-siRNA, levels were quantified by summing 21-nt small RNA reads that mapped to each of the eight *TAS* loci.

### Immunoprecipitation and protein gel blot analysis

2 g of 12-day-old seedlings was ground into fine powder in liquid nitrogen and the powder was homogenized in 3 ml lysis buffer (50 mM Tris-HCl, pH7.5, 150 mM NaCl, 10% glycerol, 0.1% CA-630, one tablet of cOmplete EDTA-free Protease Inhibitor Cocktail /50ml (Roche)), and incubated for 30 min at 4°C with gentle rotation. The total lysate was centrifuged at 12,000 g at 4°C for 20 min twice to remove cell debris. Meanwhile, 30 μl Dynabeads Protein A (ThermoFisher) was incubated with 8 μg of anti-HA antibody (Sigma, H6908) for 30 min at room temperature with gentle rotation, and the beads were then washed with lysis buffer 5 times to remove the extra antibody. The lysate was incubated with the anti-HA antibody-protein A beads for 2 hours at 4°C with gentle rotation, and then beads were captured magnetically and washed with lysis buffer 5 times. Washed beads were divided for small RNA and protein analysis.

For small RNA analysis, washed beads were boiled in H_2_O for 5 min under constant shaking and removed magnetically. The supernatant containing immunoprecipitated RNA was used in small RNA library construction (as described above). For protein gel blot analysis, washed beads were boiled in 2xSDS sample buffer (50 mM Tris-HCL at pH 6.8, 10% glycerol, 2% SDS, 0.1% bromophenol blue, and 1% 2-mercaptoethanol) for 10 min followed by vigorous shaking. The beads were then removed magnetically, and the supernatant containing the immunoprecipitated protein was resolved in a 10% SDS-PAGE gel and detected by an anti-HA antibody (Roche, 12158167001).

### RNA-seq and data analysis

Total RNA was isolated from 12-day-old Arabidopsis seedlings using TRI Reagent (NRC), and DNA was removed by DNase I (Roche) treatment. PolyA RNA was then enriched from 1 μg of DNase I-treated RNA using Oligo d(T)25 Magnetic Beads (NEB S1419S), followed by RNA-seq libraries construction using the NEBNext Ultra Directional RNA Library Prep Kit for Illumina (NEB E7420). RNA libraries were sequenced on an Illumina NovaSeq 6000 platform (PE150 bp), and the sequencing data were analyzed using the pRNASeqTools v.0.8 pipeline. Briefly, raw reads were aligned to the Arabidopsis genome (TAIR 10) using STAR v2.7.6a [56] with parameters ‘—alignIntronMax 5000—outSAMmultNmax 1—outFilterMultimapNmax 50—outFilterMismatchNoverLmax 0.1’, and counted by featureCounts v2.0.0 [57]. Normalization was performed by calculating the FPKM (Fragments Per Kilobase Million) for each gene, and differential gene expression analysis was conducted by DESeq2 v1.30.0 with a fold change of 1.5 and adjusted *P* value < 0.05 as the parameters [55].

### Small RNA phasing analysis

Small RNA phasing analysis was performed as previously described [10,20,58]. Small RNA reads from both the sense and antisense strands were included in the analysis. The formula used for phasing score calculation was described before [10].

### Nuclear–cytoplasmic fractionation

Nuclear-cytoplasmic fractionation was performed as described [37]. 1 g of 12-day-old Arabidopsis seedlings was crosslinked in 0.5% formaldehyde/1× PBS buffer through vacuum infiltration for 15 min at room temperature, and crosslinking was stopped by vacuum infiltration in 100 mM glycine/1× PBS buffer for 5 min at room temperature. The plant material was washed with 1× PBS buffer and blotted dry, and then ground to a fine powder in liquid nitrogen. The powder was resuspended in 2 ml lysis buffer (20 mM Tris-HCl, pH7.5, 20 mM KCl, 2.5 mM MgCl_2_, 2 mM EDTA, 25% glycerol, 250 mM sucrose, 5 mM DTT and cOmplete Protease Inhibitor Cocktail (Roche)) and filtered through a 40 μm cell strainer (Falcon). The flow-through was centrifuged at 1,500g for 10 min at 4°C. The supernatant representing the cytoplasmic fraction was further centrifuged at 10,000g for 10 min at 4°C to remove any residual debris. The pellet from the 1,500g spin representing the nuclei was dissolved with 10 ml nuclei resuspension buffer 1 (NRB1) (20 mM Tris-HCl, pH7.4, 2.5 mM MgCl_2_, and 0.2% Triton X-100) and centrifuged at 1,500g for 10 min to collect nuclei; this step was repeated 8 times to thoroughly wash the nuclei. After the final wash, the pellet was resuspended with 500 μl NRB2 (20 mM Tris-HCl, pH7.5, 250 mM sucrose, 10 mM MgCl_2_, 0.5% Triton X-100, 5 mM 2-mercaptoethanol, and cOmplete Protease Inhibitor Cocktail (Roche)), and carefully loaded onto 500 μl NRB3 (20 mM Tris-HCl, pH7.5, 1.7 M sucrose, 10 mM MgCl_2_, 0.5% Triton X-100, 5 mM 2-mercaptoethanol, and cOmplete Protease Inhibitor Cocktail (Roche)) without disturbing the bottom layer, then the sample was centrifuged at 16,000g for 45 min at 4°C. The nuclear pellet was resuspended and boiled in 1XSDS loading buffer for 10 min for protein gel blot analysis.

### Multiple sequence alignment

Protein sequences of AGO1 paralogs and orthologs from *Arabidopsis thaliana* and representative species were acquired from Uniprot (https://www.uniprot.org/), including 10 *Arabidopsis thaliana* AGOs, AGO1 (O04379), AGO2 (Q9SHF3), AGO3 (Q9SHF2), AGO4 (Q9ZVD5), AGO5 (Q9SJK3), AGO6 (O48771), AGO7 (Q9C793), AGO8 (Q3E984), AGO9 (Q84VQ0), and AGO10 (Q9XGW1), *Arabidopsis lyrata* AGO1 (D7KD09), *Brassica napus* AGO1 (A0A078JMZ3), *Glycine max* AGO1a (I1MQL3), *Oryza sativa* AGO1a (Q6EU14), and *Zea mays* AGO1a (A0A096TTL7). Protein sequence alignments were conducted by MUSCLE [59] (https://www.ebi.ac.uk/Tools/msa/muscle/) with default settings, and figures were made using ESPript3 [60] (https://espript.ibcp.fr).

### Structure prediction

The protein sequence of *Arabidopsis thaliana* AGO1 (O04379) was obtained from Uniprot (https://www.uniprot.org/). Structure prediction was conducted by AlphaFold [61] (https://alphafold.ebi.ac.uk/) with default settings. Protein structure homology-modelling was performed by SWISS-MODEL (https://swissmodel.expasy.org/).

### Protein feature analysis

Protein domain and motif analysis was performed by Pfam (http://pfam.xfam.org/), InterPro (https://www.ebi.ac.uk/interpro/), and NCBI Conserved Domains Database (https://www.ncbi.nlm.nih.gov/Structure/cdd/wrpsb.cgi. Protein subcellular localization prediction was performed by WoLFPSORT (https://wolfpsort.hgc.jp/), SeqNLS (http://mleg.cse.sc.edu/seqNLS/), cNLS Mapper (http://nls-mapper.iab.keio.ac.jp/cgi-bin/NLS_Mapper_form.cgi), and NESmapper (https://sourceforge.net/projects/nesmapper/).

### Correlation analysis

Pearson’s correlation coefficients (*R*) and p-values were calculated in R (v4.2.2) with the function cor.test(x,y).

## Supporting information

S1 Fig Phylogenetic analysis of Arabidopsis AGOs and Arabidopsis AGO NTEs, and sequence alignment of Arabidopsis AGO NTEs. **(A)** Phylogenetic analysis of 10 full-length Arabidopsis AGOs. The black bars denote the lengths of the NTE regions in the AGOs. **(B)** Alignments showing the conservation or lack of conservation of the NTE regions in the Arabidopsis AGO family. The red rectangle denotes amino acid identity in all proteins. Yellow rectangles denote similar residues. The AGO1 NLS and NES are marked by the red lines. **(C)** Phylogenetic analysis of the NTE regions of 10 Arabidopsis AGOs.
(TIF)

S2 Fig Sequence alignments showing the conservation or lack of conservation of *Arabidopsis thaliana* AGO1 NTE regions in AGO1s from six plant species, including *Arabidopsis thaliana* (*At*), *Arabidopsis lyrate* (*Al*), *Brassica napus* (*Bn*), *Glycine max* (*Gm*), *Oryza sativa* (*Os*), and *Zea mays* (*Zm*). The red rectangles denote amino acid identity in all proteins. Yellow rectangles denote similar residues. The NLS and NES of *Arabidopsis thaliana* AGO1 are marked by the red lines.
(TIF)

S3 Fig Western blots to determine the protein levels of wild-type AGO1 and AGO1 NTE mutants from transgenic lines expressing various transgenes in *ago1-36*. The HA antibody was used to detect HA-AGO1 and HA-AGO1 mutants. GAPDH is a loading control. The numbers within the blots indicate the relative levels of AGO1 proteins. Transgenic lines highlighted in red were selected for analysis in this study.
(TIF)

S4 Fig Analysis of small RNA-seq data of AGO1 NTE mutants. **(A)** PCA showing the reproducibility of the three replicates for each genotype. **(B)** Size (in nucleotides (nt)) distribution depicting the abundance of 18- to 26-nt total small RNAs and miRNAs in *ago1-36* expressing AGO1 and AGO1 NTE-truncated mutants. RPM, reads per million (see Methods).
(TIF)

S5 Fig Analysis of small RNA-seq data of AGO1-associated small RNAs. **(A)** PCA analysis showing that the two biological replicates of each genotype were reproducible. **(B)** Bar plots showing the composition of reads corresponding to annotated 19-to-24 nt miRNAs in AGO1 IPs and AGO1Δ1–90 IPs. Reads mapping to miRNA*s were excluded from this analysis. Annotated 20-nt miRNAs showed increased association with AGO1Δ1–90 as compared to wild-type AGO1. **(C)** Scatter plots comparing the log_2_(fold change) of IP-ed miRNAs and miRNA*s between pairs of AGO1 mutants. Pearson’s correlation coefficients (*R*) and *p*-values are indicated for miRNAs and miRNA*s separately. **(D)** Heatmap depicting the levels of IP-ed miRNAs and their corresponding miRNA*s in AGO1 mutants. Note that only miRNAs for which the corresponding miRNA*s also passed the abundance filter (average RPM > 2) are included in the heatmap.
(TIF)

S6 Fig The N-terminal extension of AGO1 allows AGO1 to distinguish between Dicer-dependent small RNAs and rRNA-derived small RNAs (rsRNAs). **(A to C)** Analysis of small RNA-seq data of AGO1-associated small RNAs. Wild-type AGO1 and AGO1Δ1–90 were in the *ago1-36* background, while other AGO1 NTE-truncated mutants were in the *ago1-36/+* background. Two biological replicates of each genotype were included in the analysis. **(A)** Composition of small RNAs in the 20-, 21-, 22-, and 24-nt classes in IPs from wild-type AGO1 (WT) and AGO1 NTE-truncated forms. Each column represents a biological replicate. **(B)** Size (in nucleotides (nt)) distribution depicting the abundance of 18- to 26-nt rRNA-derived small RNAs (rsRNAs) in IPs from AGO1 and AGO1 NTE-truncated mutants. RPM, reads per million (see Methods). **(C)** Bar plot depicting the composition of 5’ terminal nucleotides of small RNAs bound by AGO1 and AGO1 NTE-truncated mutants. The X axis represents the size of small RNAs.
(TIF)

S7 Fig Western blots to determine the nuclear-cytoplasmic partitioning of AGO1 in AGO1 NTE mutants. Blots were probed using AGO1 or HA antibodies to detect AGO1. Histone H3 is a nuclear marker and used to quantify AGO1 in the total lysate (T) and the nuclear fraction (N). cFBPase is a cytoplasmic marker and used to quantify AGO1 in the T and the cytoplasmic fraction (C). The numbers represent relative protein levels. The arrows indicate the endogenous protein (AGO1) and the protein from the transgene (HA- AGO1Δ91–189 or GFP-AGO1mNES), with the two being distinguished by size.
(TIF)

S8 Fig PCA analysis of RNA-seq data of AGO1 NTE mutants. Three biological replicates of each genotype cluster together.
(TIF)

S9 Fig Biogenesis of phasiRNAs in NTE-truncated AGO1 mutants. **(A)** The phasing of small RNAs over the *TAS* transcripts in *ago1-36* and *ago1-36* expressing wild-type *AGO1* or *AGO1* NTE mutants. **(B)** RNA gel blot analysis of ta-siRNA abundance in 12-day-old seedlings of *ago1-3*, *ago1-36*, and *ago1-36* expressing *AGO1* full-length or various NTE-truncated forms. The numbers represent ta-siRNA abundance in different genotypes relative to *AGO1 ago1-36*. The U6 blots serve as a loading control for the miRNA blots above. **(C)** The phasing (top) and the abundance (bottom) of small RNAs over *AGO1*, *PPR*, and *NBS-LRR* gene transcripts in *ago1-36* and *ago1-36* expressing wild-type *AGO1* or *AGO1* NTE mutants. **(D)** Bar plots showing the log_2_(fold change) of RNAs from *TAS* loci and several protein-coding genes between *ago1-36*, *ago1-36* expressing various *AGO1* mutants, and *AGO1 ago1-36*. Blue bars denote RNA with significantly different expression (fold change > 1.5 and adjust *P* value < 0.01).
(TIF)

S1 Table List of differentially accumulated small RNAs in AGO1 mutants. (CSV)

S2 Table List of differentially accumulated AGO1-IPed small RNAs. (CSV)

S3 Table RNA-Seq results in AGO1 mutants. (CSV)

S4 Table Primers and probes used in this study. (XLSX)

## References

[1] Yu Y, Zhang Y, Chen X, Chen Y. Plant noncoding RNAs: hidden players in development and stress responses. Annu Rev Cell Dev Biol. 2019;35:407–31. doi: 10.1146/annurev-cellbio-100818-125218 31403819 PMC8034839

[2] Song X, Li Y, Cao X, Qi Y. MicroRNAs and their regulatory roles in plant-environment interactions. Annu Rev Plant Biol. 2019;70:489–525. doi: 10.1146/annurev-arplant-050718-100334 30848930

[3] Xie Z, Allen E, Fahlgren N, Calamar A, Givan SA, Carrington JC. Expression of Arabidopsis MIRNA genes. Plant Physiol. 2005;138(4):2145–54. doi: 10.1104/pp.105.062943 16040653 PMC1183402

[4] Park W, Li J, Song R, Messing J, Chen X. CARPEL FACTORY, a Dicer homolog, and HEN1, a novel protein, act in microRNA metabolism in Arabidopsis thaliana. Curr Biol. 2002;12(17):1484–95. doi: 10.1016/s0960-9822(02)01017-5 12225663 PMC5137372

[5] Yu Y, Jia T, Chen X. The ‘how’ and ‘where’ of plant microRNAs. New Phytol. 2017;216(4):1002–17. doi: 10.1111/nph.14834 29048752 PMC6040672

[6] Yu B, Yang Z, Li J, Minakhina S, Yang M, Padgett RW, et al. Methylation as a crucial step in plant microRNA biogenesis. Science. 2005;307(5711):932–5. doi: 10.1126/science.1107130 15705854 PMC5137370

[7] Baumberger N, Baulcombe DC. Arabidopsis ARGONAUTE1 is an RNA slicer that selectively recruits microRNAs and short interfering RNAs. Proc Natl Acad Sci U S A. 2005;102(33):11928–33. doi: 10.1073/pnas.0505461102 16081530 PMC1182554

[8] Mi S, Cai T, Hu Y, Chen Y, Hodges E, Ni F, et al. Sorting of small RNAs into Arabidopsis Argonaute complexes is directed by the 5’ terminal nucleotide. Cell. 2008;133(1):116–27. doi: 10.1016/j.cell.2008.02.034 18342361 PMC2981139

[9] Li S, Liu L, Zhuang X, Yu Y, Liu X, Cui X, et al. MicroRNAs inhibit the translation of target mRNAs on the endoplasmic reticulum in Arabidopsis. Cell. 2013;153(3):562–74. doi: 10.1016/j.cell.2013.04.005 23622241 PMC3694718

[10] Li S, Le B, Ma X, Li S, You C, Yu Y, et al. Biogenesis of phased siRNAS on membrane-bound polysomes in Arabidopsis. Elife. 2016 Dec 12;5:e22750. doi: 10.7554/eLife.22750 27938667 PMC5207768

[11] Liu Y, Teng C, Xia R, Meyers BC. PhasiRNAs in plants: their biogenesis, genic sources, and roles in stress responses, development, and reproduction. Plant Cell. 2020;32(10):3059–80. doi: 10.1105/tpc.20.00335 32817252 PMC7534485

[12] Yoshikawa M, Peragine A, Mee YP, Poethig RS. A pathway for the biogenesis of trans-acting siRNAs in Arabidopsis. Genes Dev. 2005 Sep 15;19(18):2164–75. doi: 10.1101/gad.1352605 16131612 PMC1221887

[13] Gasciolli V, Mallory AC, Bartel DP, Vaucheret H. Partially redundant functions of arabidopsis DICER-like enzymes and a role for DCL4 in producing trans-acting siRNAs. Curr Biol. 2005;15(16):1494–500. doi: 10.1016/j.cub.2005.07.024 16040244

[14] Allen E, Xie Z, Gustafson AM, Carrington JC. microRNA-directed phasing during trans-acting siRNA biogenesis in plants. Cell. 2005;121(2):207–21. doi: 10.1016/j.cell.2005.04.004 15851028

[15] Montgomery TA, Howell MD, Cuperus JT, Li D, Hansen JE, Alexander AL, et al. Specificity of ARGONAUTE7-miR390 interaction and dual functionality in TAS3 trans-acting siRNA formation. Cell. 2008;133(1):128–41. doi: 10.1016/j.cell.2008.02.033 18342362

[16] Chen HM, Chen LT, Patel K, Li YH, Baulcombe DC, Wu SH. 22-nucleotide RNAs trigger secondary siRNA biogenesis in plants. Proc Natl Acad Sci U S A. 2010;107(34):15269–74. doi: 10.1073/pnas.1001738107 20643946 PMC2930544

[17] Cuperus JT, Carbonell A, Fahlgren N, Garcia-Ruiz H, Burke RT, Takeda A, et al. Unique functionality of 22-nt miRNAs in triggering RDR6-dependent siRNA biogenesis from target transcripts in Arabidopsis. Nat Struct Mol Biol. 2010;17(8):997–1003. doi: 10.1038/nsmb.1866 20562854 PMC2916640

[18] Axtell MJ, Jan C, Rajagopalan R, Bartel DP. A Two-hit trigger for siRNA biogenesis in plants. Cell. 2006;127(3):565–77. doi: 10.1016/j.cell.2006.09.032 17081978

[19] Boccara M, Sarazin A, Thiébeauld O, Jay F, Voinnet O, Navarro L, et al. The Arabidopsis miR472-RDR6 silencing pathway modulates PAMP- and effector-triggered immunity through the post-transcriptional control of disease resistance genes. PLoS Pathog. 2014;10(1):e1003883. doi: 10.1371/journal.ppat.1003883 24453975 PMC3894208

[20] Howell MD, Fahlgren N, Chapman EJ, Cumbie JS, Sullivan CM, Givan SA, et al. Genome-wide analysis of the RNA-DEPENDENT RNA POLYMERASE6/DICER-LIKE4 pathway in Arabidopsis reveals dependency on miRNA- and tasiRNA-directed targeting. Plant Cell. 2007;19(3):926–42. doi: 10.1105/tpc.107.050062 17400893 PMC1867363

[21] Kwak PB, Tomari Y. The N domain of Argonaute drives duplex unwinding during RISC assembly. Nat Struct Mol Biol. 2012;19(2):145–52. doi: 10.1038/nsmb.2232 22233755

[22] Lingel A, Simon B, Izaurralde E, Sattler M. Nucleic acid 3′-end recognition by the Argonaute2 PAZ domain. Nat Struct Mol Biol. 2004;11(6):576–7. doi: 10.1038/nsmb777 15156196

[23] Ma JB, Ye K, Patel DJ. Structural basis for overhang-specific small interfering RNA recognition by the PAZ domain. Nature. 2004;429(6989):318–22. doi: 10.1038/nature02519 15152257 PMC4700412

[24] Frank F, Sonenberg N, Nagar B. Structural basis for 5′-nucleotide base-specific recognition of guide RNA by human AGO2. Nature. 2010;465(7299):818–22. doi: 10.1038/nature09039 20505670

[25] Frank F, Hauver J, Sonenberg N, Nagar B. Arabidopsis Argonaute MID domains use their nucleotide specificity loop to sort small RNAs. EMBO J. 2012;31(17):3588–95. doi: 10.1038/emboj.2012.204 22850669 PMC3433783

[26] Song J joon, Smith SK, Hannon GJ, Joshua-Tor L. Crystal structure of Argonaute and its implications for RISC slicer activity. Science. 2004;305(5689):1434–7. doi: 10.1126/science.1102514 15284453

[27] Liu J, Carmell MA, Rivas F V., Marsden CG, Thomson JM, Song JJ, et al. Argonaute2 is the catalytic engine of mammalian RNAi. Science. 2004;305(5689):1437–41. doi: 10.1126/science.1102513 15284456

[28] Carbonell A, Fahlgren N, Garcia-Ruiz H, Gilbert KB, Montgomery TA, Nguyen T, et al. Functional analysis of three Arabidopsis ARGONAUTES using slicer-defective mutants. Plant Cell. 2012;24(9):3613–29. doi: 10.1105/tpc.112.099945 23023169 PMC3480291

[29] Qi Y, He X, Wang XJ, Kohany O, Jurka J, Hannon GJ. Distinct catalytic and non-catalytic roles of ARGONAUTE4 in RNA-directed DNA methylation. Nature. 2006;443:1008–12. doi: 10.1038/nature05198 16998468

[30] Ji L, Liu X, Yan J, Wang W, Yumul RE, Kim YJ, et al. ARGONAUTE10 and ARGONAUTE1 regulate the termination of floral stem cells through two microRNAs in Arabidopsis. PLoS Genet. 2011;7(3):1–14. doi: 10.1371/journal.pgen.1001358 21483759 PMC3069122

[31] Schirle NT, MacRae IJ. The crystal structure of Human Argonaute2. Science. 2012;336(1):1037–40. doi: 10.1126/science.1221551 22539551 PMC3521581

[32] Nakanishi K, Weinberg DE, Bartel DP, Patel DJ. Structure of yeast Argonaute with guide RNA. Nature. 2012;486:368–74. doi: 10.1038/nature11211 22722195 PMC3853139

[33] Elkayam E, Kuhn CD, Tocilj A, Haase AD, Greene EM, Hannon GJ, et al. The structure of human argonaute-2 in complex with miR-20a. Cell. 2012;150(1):100–10. doi: 10.1016/j.cell.2012.05.017 22682761 PMC3464090

[34] Wang Y, Sheng G, Juranek S, Tuschl T, Patel DJ. Structure of the guide-strand-containing argonaute silencing complex. Nature. 2008;456(November):209–14. doi: 10.1038/nature07315 18754009 PMC4689319

[35] Poulsen C, Vaucheret H, Brodersen P. Lessons on RNA silencing mechanisms in plants from eukaryotic Argonaute structures. Plant Cell. 2013;25(1):22–37. doi: 10.1105/tpc.112.105643 23303917 PMC3584537

[36] Bologna NG, Iselin R, Abriata LA, Sarazin A, Pumplin N, Jay F, et al. Nucleo-cytosolic shuttling of ARGONAUTE1 prompts a revised model of the plant microRNA pathway. Mol Cell. 2018;69(4):709–719.e5. doi: 10.1016/j.molcel.2018.01.007 29398448

[37] Zhang B, You C, Zhang Y, Zeng L, Hu J, Zhao M, et al. Linking key steps of microRNA biogenesis by TREX-2 and the nuclear pore complex in Arabidopsis. Nat Plants. 2020;6(8):957–69. doi: 10.1038/s41477-020-0726-z 32690891 PMC7426256

[38] Fan L, Zhang C, Gao B, Zhang Y, Stewart E, Jez J, et al. Microtubules promote the non-cell autonomous action of microRNAs by inhibiting their cytoplasmic loading onto ARGONAUTE1 in Arabidopsis. Dev Cell. 2022;57(8):995–1008.e1-e5. doi: 10.1016/j.devcel.2022.03.015 35429434 PMC9056376

[39] Gregory BD, O’Malley RC, Lister R, Urich MA, Tonti-Filippini J, Chen H, et al. A link between RNA metabolism and silencing affecting Arabidopsis development. Dev Cell. 2008;14(6):854–66. doi: 10.1016/j.devcel.2008.04.005 18486559

[40] Brodersen P, Sakvarelidze-Achard L, Schaller H, Khafif M, Schott G, Bendahmane A, et al. Isoprenoid biosynthesis is required for miRNA function and affects membrane association of ARGONAUTE 1 in Arabidopsis. Proc Natl Acad Sci U S A. 2012;109(5):1778–83. doi: 10.1073/pnas.1112500109 22247288 PMC3277166

[41] Vaucheret H, Mallory AC, Bartel DP. AGO1 homeostasis entails coexpression of MIR168 and AGO1 and preferential stabilization of miR168 by AGO1. Mol Cell. 2006;22(1):129–36. doi: 10.1016/j.molcel.2006.03.011 16600876 PMC2323247

[42] Bressendorff S, Sjøgaard IMZ, Oksbjerg ED, Kausika S, Michels A, Prestel A, et al. Distinction between small RNA-bound and free ARGONAUTE via an N-terminal protein-protein interaction site. bioRxiv. 2022;2022.10.22.513346.

[43] Bressendorff S, Kausika S1, Sjøgaard IMZ, Oksbjerg ED, Michels A, Poulsen C, et al. The N-coil and the globular N-terminal domain of plant ARGONAUTE1 are interaction hubs for regulatory factors. bioRxiv. 2023;2023.01.18.524620. 10.1042/BCJ20230025 37278687

[44] Gudipati RK, Braun K, Gypas F, Hess D, Schreier J, Carl SH, et al. Protease-mediated processing of Argonaute proteins controls small RNA association. Mol Cell. 2021;81(11):2388–402. doi: 10.1016/j.molcel.2021.03.029 33852894

[45] Meyer WJ, Schreiber S, Guo Y, Volkmann T, Welte MA, Müller HAJ. Overlapping functions of Argonaute proteins in patterning and morphogenesis of Drosophila embryos. PLoS Genet. 2006;2(8):1224–39. doi: 10.1371/journal.pgen.0020134 16934003 PMC1557783

[46] Vaucheret H. Plant ARGONAUTES. Trends Plant Sci. 2008 Jul;13(7):350–8. doi: 10.1016/j.tplants.2008.04.007 18508405

[47] Thandapani P, O’Connor TR, Bailey TL, Richard S. Defining the RGG/RG motif. Mol Cell. 2013;50(5):613–23. doi: 10.1016/j.molcel.2013.05.021 23746349

[48] Bohmert K, Camus I, Bellini C, Bouchez D, Caboche M, Banning C. AGO1 defines a novel locus of Arabidopsis controlling leaf development. EMBO J. 1998;17(1):170–80. doi: 10.1093/emboj/17.1.170 9427751 PMC1170368

[49] Arribas-Hernández L, Kielpinski LJ, Brodersen P. mRNA decay of most Arabidopsis miRNA targets requires slicer activity of AGO1. Plant Physiol. 2016;171:2620–32. doi: 10.1104/pp.16.00231 27208258 PMC4972266

[50] Iwasaki S, Kobayashi M, Yoda M, Sakaguchi Y, Katsuma S, Suzuki T, et al. Hsc70/Hsp90 chaperone machinery mediates ATP-dependent RISC loading of small RNA duplexes. Mol Cell. 2010;39(2):292–9. doi: 10.1016/j.molcel.2010.05.015 20605501

[51] Nakamura S, Mano S, Tanaka Y, Ohnishi M, Nakamori C, Araki M, et al. Gateway binary vectors with the bialaphos resistance gene, bar, as a selection marker for plant transformation. Biosci Biotechnol Biochem. 2010;74(6):1315–9. doi: 10.1271/bbb.100184 20530878

[52] Earley KW, Haag JR, Pontes O, Opper K, Juehne T, Song K, et al. Gateway-compatible vectors for plant functional genomics and proteomics. Plant J. 2006;45(4):616–29. doi: 10.1111/j.1365-313X.2005.02617.x 16441352

[53] Martin M. Cutadapt removes adapter sequences from high-throughput sequencing reads. EMBnet.journal. 2011;17(1):10.

[54] Johnson NR, Yeoh JM, Coruh C, Axtell MJ. Improved placement of multi-mapping small RNAs. G3 Genes, Genomes, Genet. 2016;6(7):2103–11. 10.1534/g3.116.030452 PMC4938663 27175019

[55] Love MI, Huber W, Anders S. Moderated estimation of fold change and dispersion for RNA-seq data with DESeq2. Genome Biol. 2014;15(550). doi: 10.1186/s13059-014-0550-8 25516281 PMC4302049

[56] Dobin A, Davis CA, Schlesinger F, Drenkow J, Zaleski C, Jha S, et al. STAR: Ultrafast universal RNA-seq aligner. Bioinformatics. 2013;29(1):15–21. doi: 10.1093/bioinformatics/bts635 23104886 PMC3530905

[57] Liao Y, Smyth GK, Shi W. FeatureCounts: An efficient general purpose program for assigning sequence reads to genomic features. Bioinformatics. 2014;30(7):923–30. doi: 10.1093/bioinformatics/btt656 24227677

[58] De Paoli E, Dorantes-Acosta A, Jixian Z, Accerbi M, Jeong DH, Sunhee P, et al. Distinct extremely abundant siRNAs associated with cosuppression in petunia. RNA. 2009;15(11):1965–70. doi: 10.1261/rna.1706109 19776157 PMC2764480

[59] Edgar RC. MUSCLE: a multiple sequence alignment method with reduced time and space complexity. BMC Bioinformatics. 2004;5(113). doi: 10.1186/1471-2105-5-113 15318951 PMC517706

[60] Robert X, Gouet P. Deciphering key features in protein structures with the new ENDscript server. Nucleic Acids Res. 2014 Jul;42:W320–4. doi: 10.1093/nar/gku316 24753421 PMC4086106

[61] Jumper J, Evans R, Pritzel A, Green T, Figurnov M, Ronneberger O, et al. Highly accurate protein structure prediction with AlphaFold. Nature. 2021;596(7873):583–9. doi: 10.1038/s41586-021-03819-2 34265844 PMC8371605

