# Supplementary figures and images for: The N-terminal extension of Arabidopsis ARGONAUTE 1 is essential for microRNA activities
Source: PLoS Genet. 2023 Mar 8;19(3):e1010450. doi: 10.1371/journal.pgen.1010450 (PMC9994745; doi:10.1371/journal.pgen.1010450)

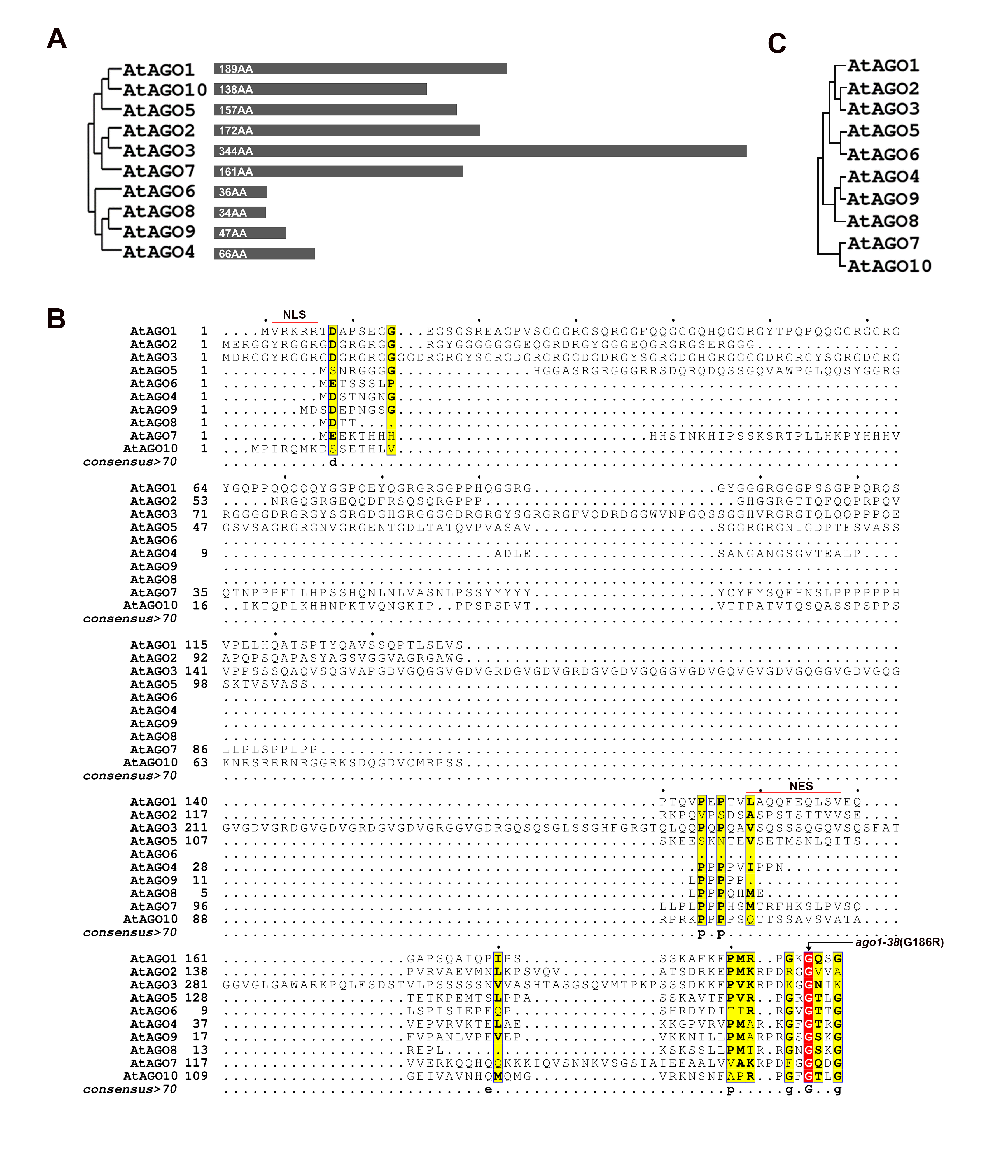

Supplement: S1 Fig — (A) Phylogenetic analysis of 10 full-length Arabidopsis AGOs. The black bars denote the lengths of the NTE regions in the AGOs. (B) Alignments showing the conservation or lack of conservation of the NTE regions in the Arabidopsis AGO family. The red rectangle denotes amino acid identity in all proteins. Yellow rectangles denote similar residues. The AGO1 NLS and NES are marked by the red lines. (C) Phylogenetic analysis of the NTE regions of 10 Arabidopsis AGOs. (TIF) [file pgen.1010450.s001.tif]

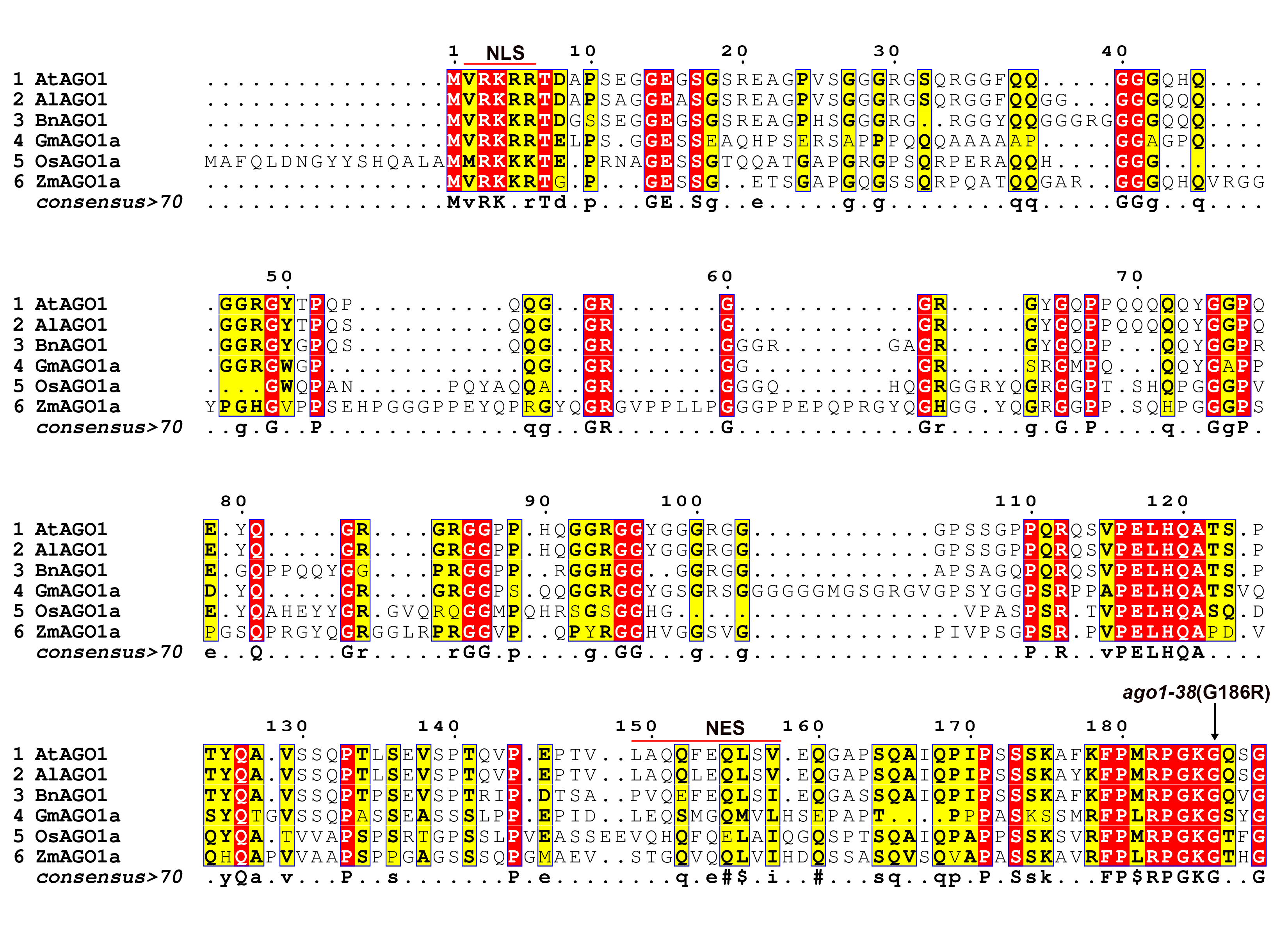

Supplement: S2 Fig — The red rectangles denote amino acid identity in all proteins. Yellow rectangles denote similar residues. The NLS and NES of Arabidopsis thaliana AGO1 are marked by the red lines. (TIF) [file pgen.1010450.s002.tif]

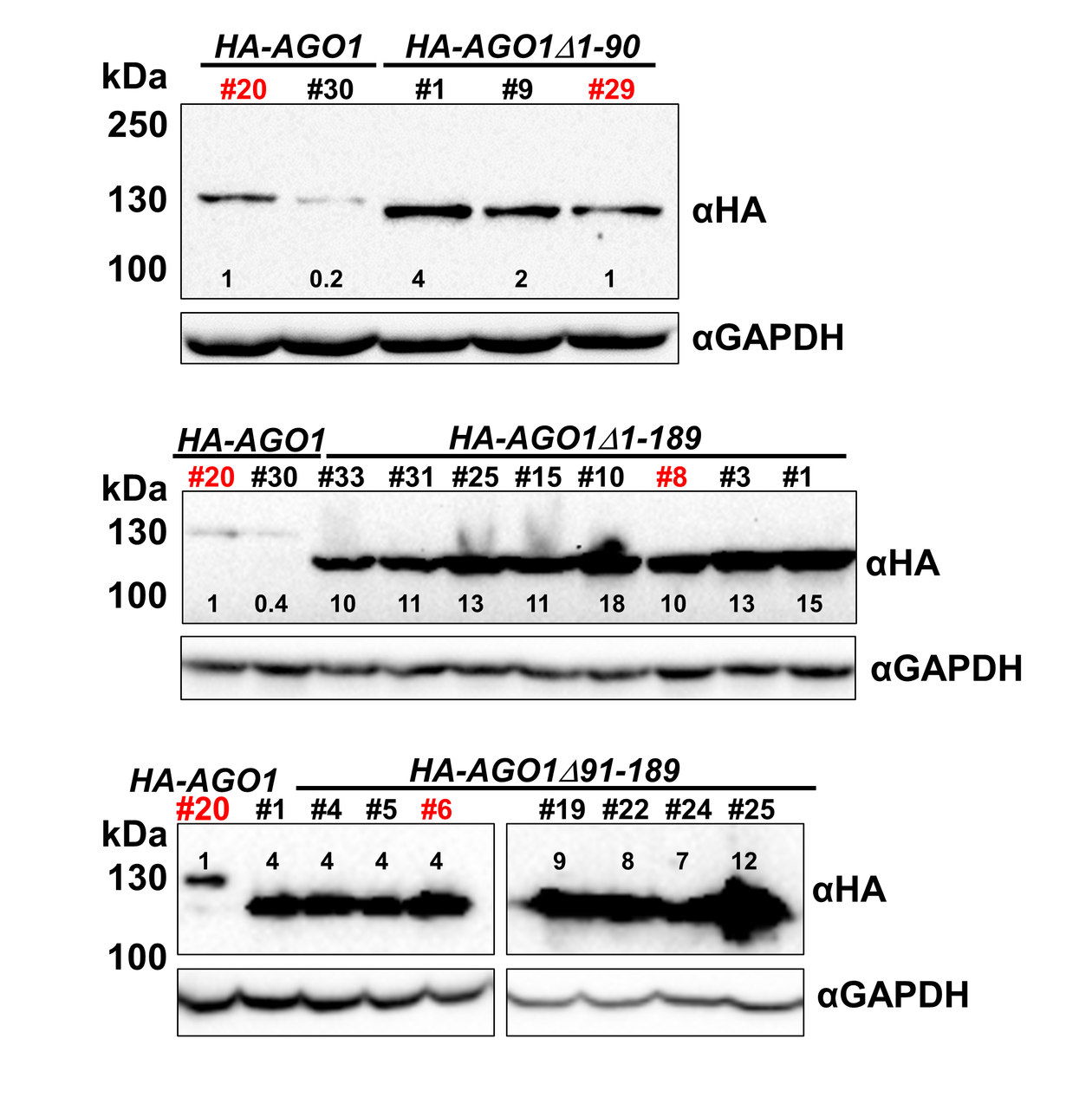

Supplement: S3 Fig — The HA antibody was used to detect HA-AGO1 and HA-AGO1 mutants. GAPDH is a loading control. The numbers within the blots indicate the relative levels of AGO1 proteins. Transgenic lines highlighted in red were selected for analysis in this study. (TIF) [file pgen.1010450.s003.tif]

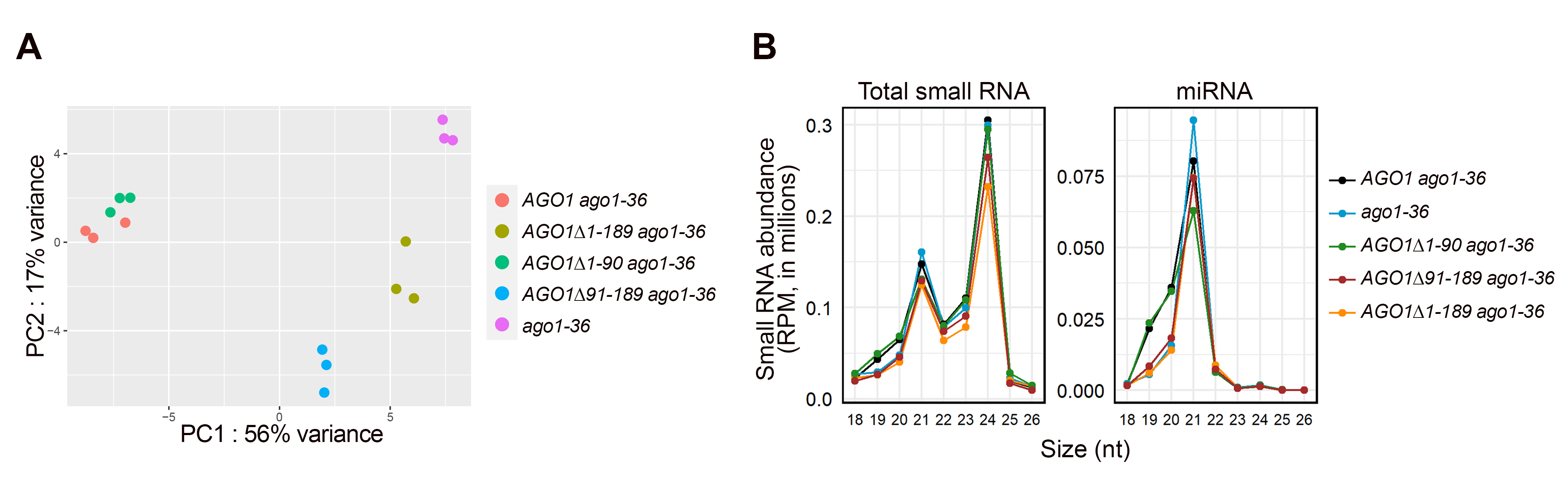

Supplement: S4 Fig — (A) PCA showing the reproducibility of the three replicates for each genotype. (B) Size (in nucleotides (nt)) distribution depicting the abundance of 18- to 26-nt total small RNAs and miRNAs in ago1-36 expressing AGO1 and AGO1 NTE-truncated mutants. RPM, reads per million (see Methods). (TIF) [file pgen.1010450.s004.tif]

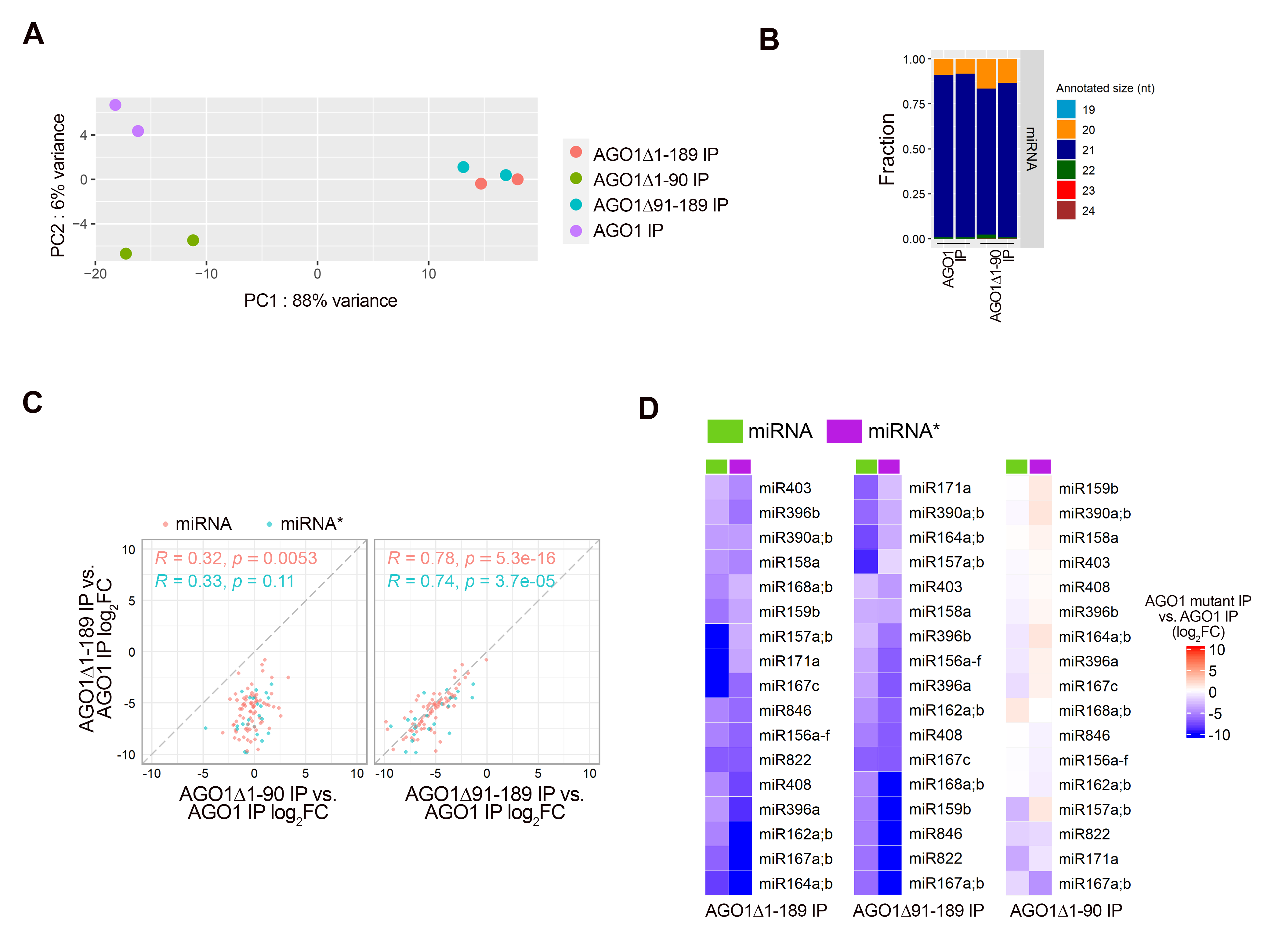

Supplement: S5 Fig — (A) PCA analysis showing that the two biological replicates of each genotype were reproducible. (B) Bar plots showing the composition of reads corresponding to annotated 19-to-24 nt miRNAs in AGO1 IPs and AGO1Δ1–90 IPs. Reads mapping to miRNA*s were excluded from this analysis. Annotated 20-nt miRNAs showed increased association with AGO1Δ1–90 as compared to wild-type AGO1. (C) Scatter plots comparing the log2(fold change) of IP-ed miRNAs and miRNA*s between pairs of AGO1 mutants. Pearson’s correlation coefficients (R) and p-values are indicated for miRNAs and miRNA*s separately. (D) Heatmap depicting the levels of IP-ed miRNAs and their corresponding miRNA*s in AGO1 mutants. Note that only miRNAs for which the corresponding miRNA*s also passed the abundance filter (average RPM > 2) are included in the heatmap. (TIF) [file pgen.1010450.s005.tif]

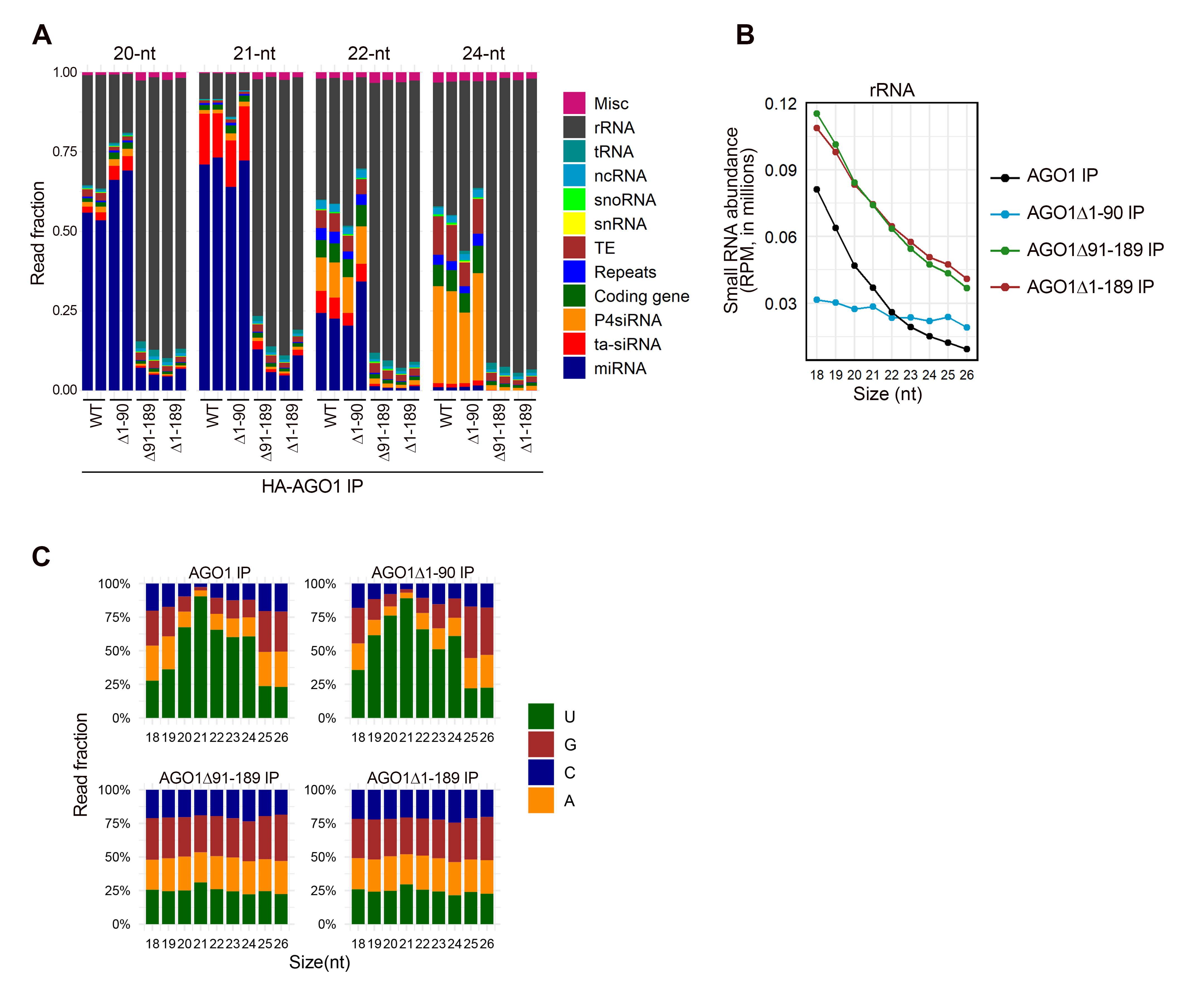

Supplement: S6 Fig — (A to C) Analysis of small RNA-seq data of AGO1-associated small RNAs. Wild-type AGO1 and AGO1Δ1–90 were in the ago1-36 background, while other AGO1 NTE-truncated mutants were in the ago1-36/+ background. Two biological replicates of each genotype were included in the analysis. (A) Composition of small RNAs in the 20-, 21-, 22-, and 24-nt classes in IPs from wild-type AGO1 (WT) and AGO1 NTE-truncated forms. Each column represents a biological replicate. (B) Size (in nucleotides (nt)) distribution depicting the abundance of 18- to 26-nt rRNA-derived small RNAs (rsRNAs) in IPs from AGO1 and AGO1 NTE-truncated mutants. RPM, reads per million (see Methods). (C) Bar plot depicting the composition of 5’ terminal nucleotides of small RNAs bound by AGO1 and AGO1 NTE-truncated mutants. The X axis represents the size of small RNAs. (TIF) [file pgen.1010450.s006.tif]

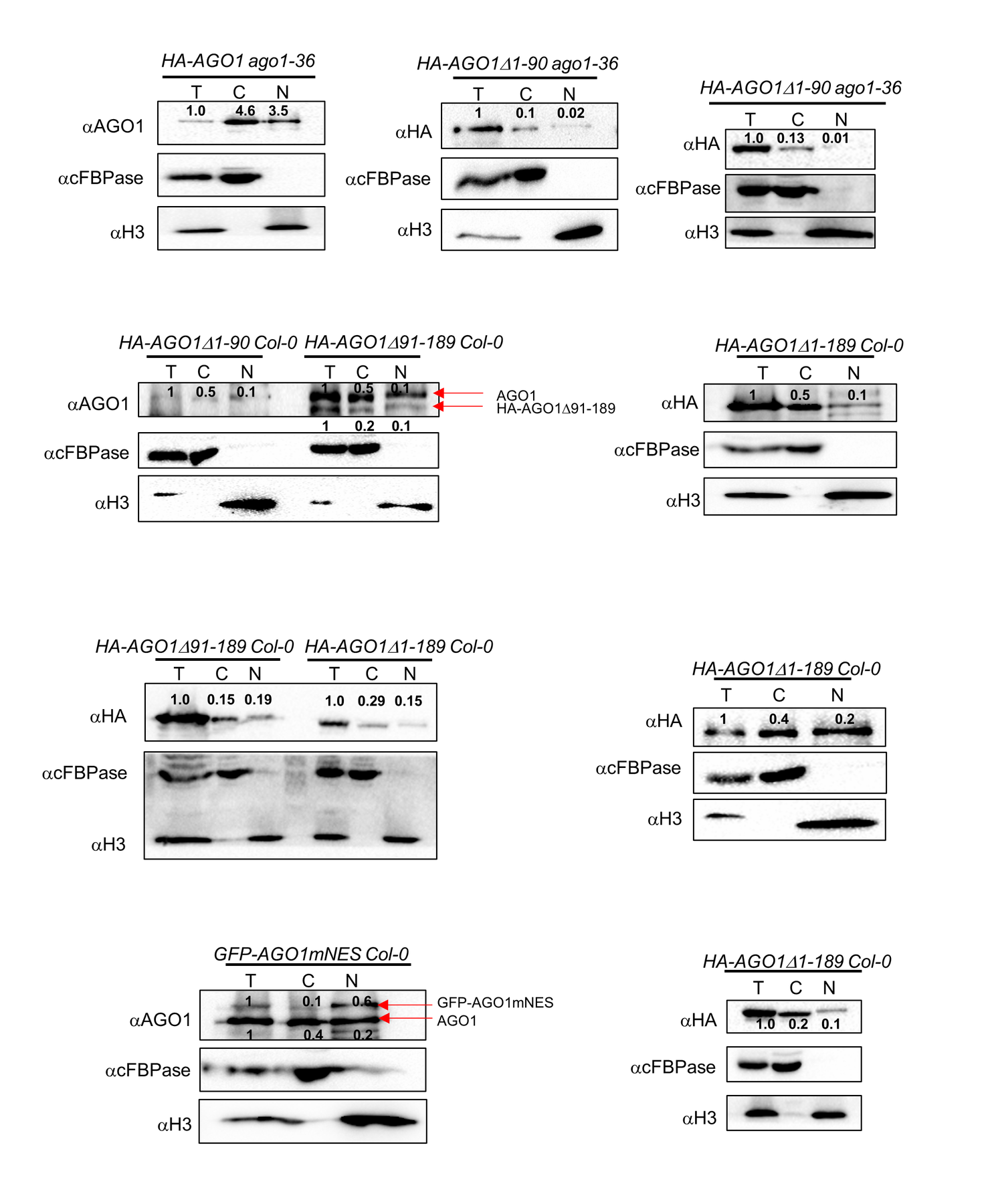

Supplement: S7 Fig — Blots were probed using AGO1 or HA antibodies to detect AGO1. Histone H3 is a nuclear marker and used to quantify AGO1 in the total lysate (T) and the nuclear fraction (N). cFBPase is a cytoplasmic marker and used to quantify AGO1 in the T and the cytoplasmic fraction (C). The numbers represent relative protein levels. The arrows indicate the endogenous protein (AGO1) and the protein from the transgene (HA- AGO1Δ91–189 or GFP-AGO1mNES), with the two being distinguished by size. (TIF) [file pgen.1010450.s007.tif]

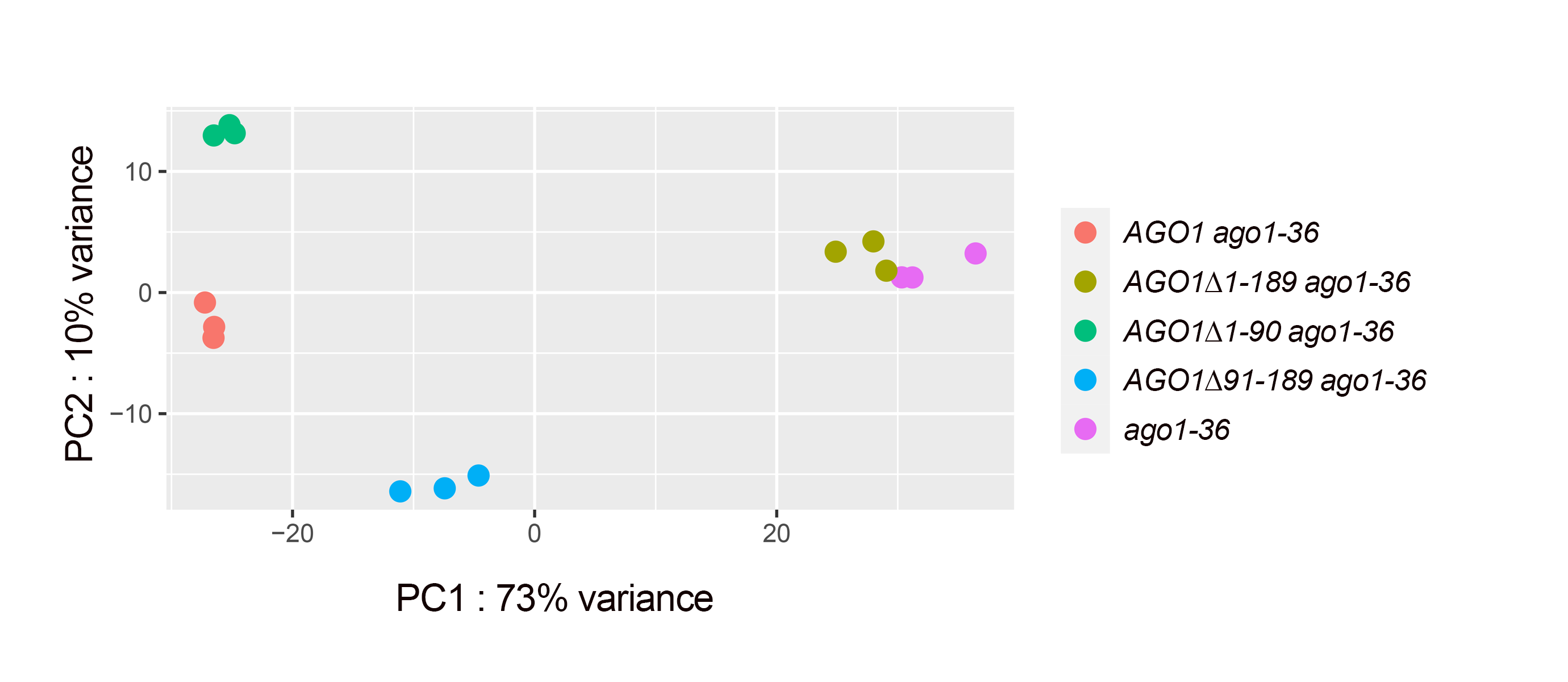

Supplement: S8 Fig — Three biological replicates of each genotype cluster together. (TIF) [file pgen.1010450.s008.tif]

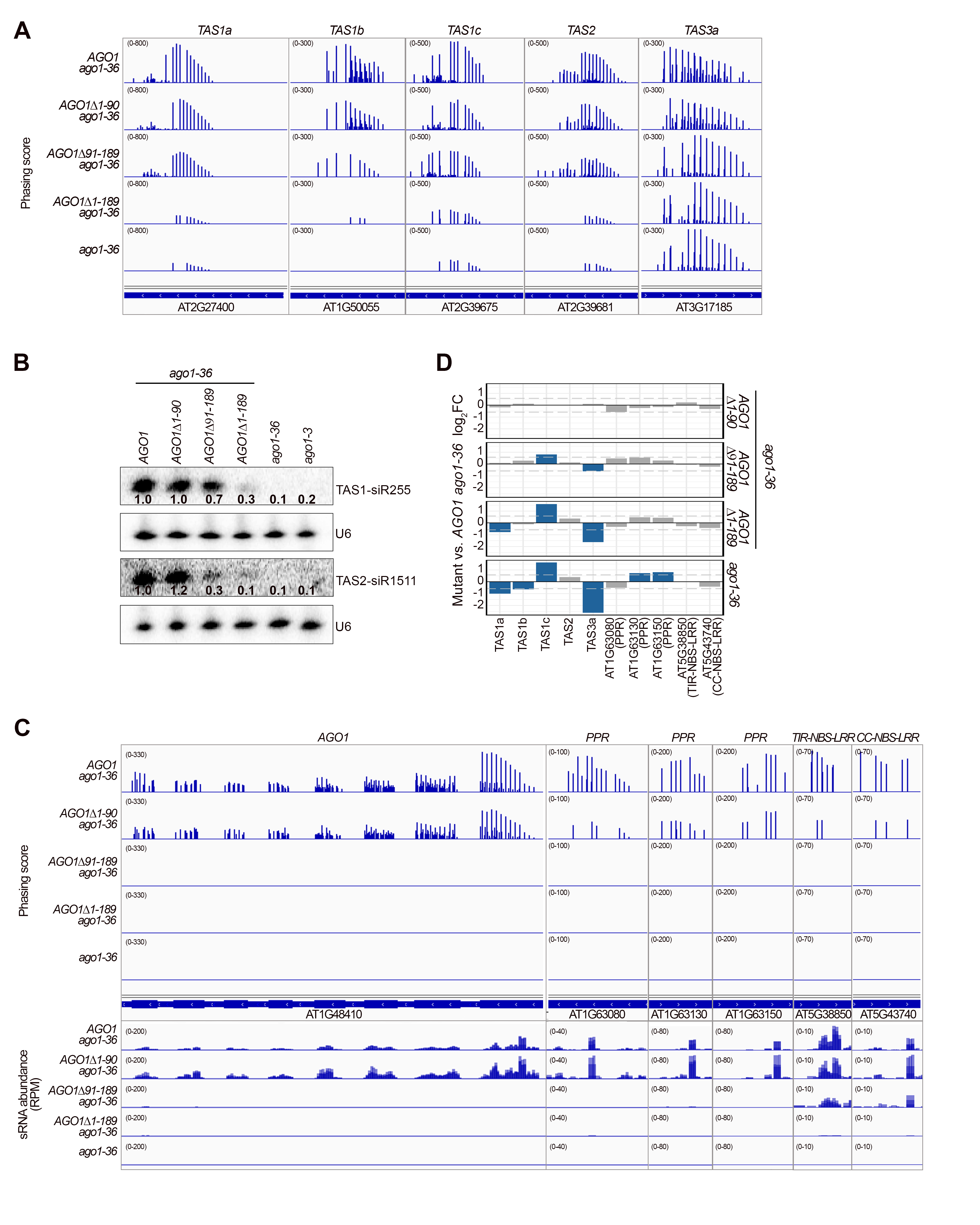

Supplement: S9 Fig — (A) The phasing of small RNAs over the TAS transcripts in ago1-36 and ago1-36 expressing wild-type AGO1 or AGO1 NTE mutants. (B) RNA gel blot analysis of ta-siRNA abundance in 12-day-old seedlings of ago1-3, ago1-36, and ago1-36 expressing AGO1 full-length or various NTE-truncated forms. The numbers represent ta-siRNA abundance in different genotypes relative to AGO1 ago1-36. The U6 blots serve as a loading control for the miRNA blots above. (C) The phasing (top) and the abundance (bottom) of small RNAs over AGO1, PPR, and NBS-LRR gene transcripts in ago1-36 and ago1-36 expressing wild-type AGO1 or AGO1 NTE mutants. (D) Bar plots showing the log2(fold change) of RNAs from TAS loci and several protein-coding genes between ago1-36, ago1-36 expressing various AGO1 mutants, and AGO1 ago1-36. Blue bars denote RNA with significantly different expression (fold change > 1.5 and adjust P value < 0.01). (TIF) [file pgen.1010450.s009.tif]
